# Decoding neddylation in malignancies: molecular mechanisms, biological functions, therapeutic resistance, and clinical potential

**DOI:** 10.1038/s41419-025-08255-y

**Published:** 2026-01-15

**Authors:** Na Deng, Qiang Sun, Xue Yu, Ting Li, Jiaxing Sun, Shiheng Jia, Shuang Ma, Weiwei Liu, Heng Zhou

**Affiliations:** 1 Key Laboratory of Molecular Pathology and Epidemiology of Gastric Cancer in Liaoning Education Department, The First Hospital of China Medical University, Shenyang, Liaoning China; 2 Department of Hematology, The Fourth Affiliated Hospital of China Medical University, Shenyang, Liaoning China; 3 Department of Plastic Surgery, The First Hospital of China Medical University, Shenyang, Liaoning China; 4 Department of Surgical Oncology and General Surgery, The First Hospital of China Medical University, Shenyang, Liaoning China; 5 Department of Neurosurgery, The First Hospital of China Medical University, Shenyang, Liaoning China; 6 Department of Ultrasound, Shengjing Hospital of China Medical University, Shenyang, Liaoning China; 7 Department of Clinical Nutrition, The First Hospital of China Medical University, Shenyang, Liaoning China; 8 Department of Anesthesiology, The First Hospital of China Medical University, Shenyang, Liaoning China

**Keywords:** Oncogenes, Tumour heterogeneity

## Abstract

Neddylation, a protein post-translational modification, regulates diverse molecular biological processes in tumors, governing protein stability, function, subcellular localization, and transcriptional activity. Thus, it plays an essential role in sustaining tumorigenicity and the hallmarks of cancer. In tumors, neddylation is triggered by various forms of cellular stress, involving hypoxia, oxidative stress, and tumor metabolites, all of which drive tumor initiation and progression. This review explores the critical regulatory mechanisms and pathological features of the neddylation cascade in terms of tumor malignant evolution and therapeutic resistance. Additionally, it examines therapeutic strategies targeting NEDD8 modification, offering novel avenues for innovative cancer treatments by disrupting this dynamic, reversible modification process.

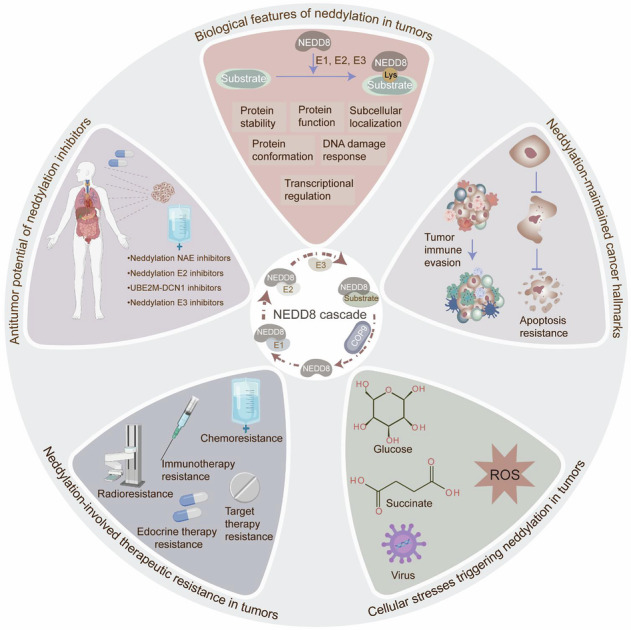

## Facts

- Neddylation is a dynamic and bidirectional post-translational modification.
- Neddylation orchestrates protein stability, protein subcellular localization, protein-protein interaction, transcriptional regulation, and protein conformation.
- Neddylation exerts a dual effect on sustaining cancer hallmarks.
- Neddylation is vital for maintaining cellular homeostasis and is triggered by various forms of cellular stress.
- Neddylation is implicated in tumor therapeutic resistance.
- Targeting neddylation offers novel avenues for innovative cancer treatments.

## Open Questions

- What is the role of neddylation in tumor initiation and progression?
- Which cellular stress can trigger neddylation?
- What is the relationship between neddylation and anti-tumor therapeutic resistance?
- Can targeting neddylation effectively inhibit tumors?

## Introduction

Neddylation, a dynamic and bidirectional post-translational modification (PTM), involves the covalent attachment of the ubiquitin-like polypeptide neural precursor cell expressed developmentally down-regulated protein 8 (NEDD8) to a specific lysine (K) residue of substrates through a three-step enzymatic cascade, modulating protein stability, subcellular localization, conformation, protein-protein interaction (PPI), and enzymatic activity [1–4]. Similar to ubiquitination, neddylation is initiated by the sequential action of NEDD8-activating enzyme E1 (NAE), NEDD8-conjugating enzyme E2s, NEDD8 E3 ligases, and terminated by deneddylases [5, 6]. Neddylation aberrations play a dual role in tumorigenic properties and cancer hallmark maintenance, such as epigenetic reprogramming, metabolic rewiring, and immune evasion, complicating its influence on tumorigenesis. Multiple cellular stress modalities, including hypoxia, high glucose conditions, and succinate accumulation, trigger neddylation dysregulation in tumors. Additionally, Neddylation drives tumor therapeutic resistance. Consequently, pharmaceutical targeting neddylation represents a promising approach in anti-tumor strategies. This review provides a comprehensive examination of neddylation in tumors, detailing its biological significance, outlining neddylation-mediated cancer hallmarks, investigating the cellular stress that activates neddylation, exploring neddylation-induced therapeutic resistance, and evaluating neddylation inhibition for cancer treatment. Recent advances in elucidating the role of neddylation in oncogenesis may provide a strong rationale for cancer treatment.

## Neddylation principles and biological features in tumors

### Overview of the Neddylation cascade

Neddylation employs a multi-enzyme cascade, involving NEDD8, NAE, NEDD8-conjugating enzyme E2s, NEDD8 E3 ligases, and deneddylases (Table 1). NEDD8, a conserved protein predominantly localized in the nucleus and widely expressed across eukaryotes [1, 7], was first identified in 1992 as an overexpressed gene in mouse neural precursor cells [8]. In 1993, it was characterized as an 81-amino-acid, 9 kDa ubiquitin-like polypeptide, exhibiting 57.8% sequence identity and 78.9% similarity to ubiquitin [9]. The conserved carboxyl-terminal Gly76 residue in the C-terminal tail of NEDD8 is a requisite for covalent attachment to substrates [1]. NAE, a significant enzyme for NEDD8 activation, is a heterodimer consisting of the regulatory subunit amyloid-beta precursor protein-binding protein 1 (APPBP1/NAE1) and the catalytic subunit ubiquitin-like modifier activating enzyme 3 (UBA3/NAEβ) [10]. E2-conjugating enzymes, including ubiquitin conjugating enzyme E2M (UBE2M/UBC12) and ubiquitin conjugating enzyme E2F (UBE2F), catalyze the transthiolation reaction [11]. Numerous NEDD8 E3 ligases, responsible for NEDD8 positioning and substrate recognition, have been ascertained, such as ring-box 1 (RBX1), RBX2, murine double minute 2 (MDM2), F-box protein 11 (FBXO11), tripartite motif 40 (TRIM40), and others [3, 7]. Deneddylases, principally including constitutive photomorphogenesis 9 (COP9) signalosome, NEDD8 protease 1 (NEDP1/DEN1/SENP8), USP21, and UCH-L3, orchestrate deneddylation by removing NEDD8 from substrates [12**–**15]. Notably, UCH-L3 and NEDP1 also facilitate the maturation of the NEDD8 precursor.

**Table 1:** Molecules and molecular characteristics in the NEDD8 pathway.

Subsets	Enzymatic activity	Molecules	Molecular weights(kDa)	Cellular location
NEDD8	None	NEDD8	9.07	Nucleus
E1	NEDD8-activating enzyme	NAE1	60.25	Cell membrane
E2	NEDD8-conjugating enzyme	UBE2M	20.9	Cytoplasm
		UBE2F	21.08	Cytoplasm, nucleus
E3	NEDD8-E3 ligases	RBX1	12.27	Cytoplasm, nucleus
		RBX2	12.68	Cytoplasm, nucleus
		MDM2/HDM2	55.23	Nucleus, cytoplasm
		TRIM	80.83	Cytoplasm
		c-CBL	52.46	Membrane
		SMURF1	86.11	Cytoplasm, membrane
		DCN1	30.12	Nucleus, cytoplasm
		cIAP1	69.9	Cytoplasm, nucleus
		FBX4	44.14	Cytoplasm
		FBXO11	103.59	Nucleus
		Hakai	54.52	Nucleus, cytoplasm
		HUWE1	481.89	Cytoplasm, nucleus, mitochondria
		Itch	102.8	Membrane, cytoplasm, nucleus
		RAD18	56.22	Nucleus, cytoplasm
		RanBP2	358.2	Nucleus
		RNF111	108.86	Nucleus, cytoplasm
		RNF168	65.02	Nucleus
		TRIM40	29.34	Cytoplasm
		XIAP	56.69	Cytoplasm, nucleus
Neddylation protease	Deneddylases	COP9	23.23	Cytoplasm, nucleus
		CSN	6.21	Nucleus, cytoplasm
		NEDP1 /DEN1	24.11	Cytoplasm
		Ataxin-3	40.75	Nucleus
		USP21	62.66	Cytoplasm, nucleus
		UCH-L1	24.82	Cytoplasm
		UCH-L3	26.18	Cytoplasm

**Fig. 1:**
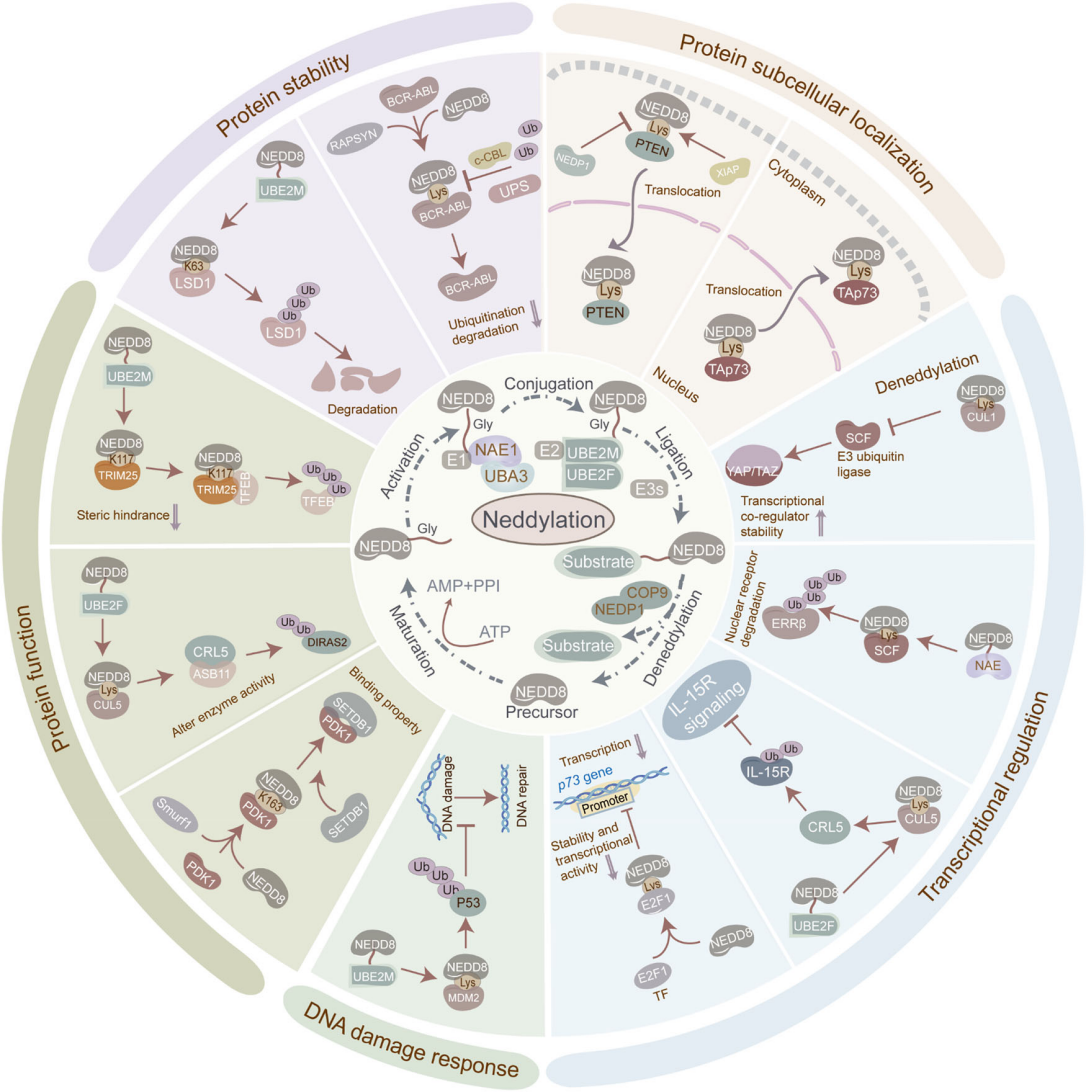
Neddylation principles and biological features in tumors. Neddylation requires the involvement of several categories of proteins, involving NEDD8, NAE, NEDD8-conjugating enzyme E2s, NEDD8 E3 ligases, and deneddylases, catalyzing NEDD8 covalently conjugating to substrate proteins. This dynamic and reversible neddylation cascade comprises multi-step actions, a succession of enzymatic reactions, involving maturation, activation, conjugation, ligation, and deneddylation. Hydrolases such as UCH-L3 and NEDP1 proteolytically cleave the C-terminal amino acids of NEDD8 precursor to expose the Gly-Gly motif, resulting in the formation of a 76-amino-acid mature NEDD8. The mature NEDD8 then interacts with the active site cysteine of NAE *via* its Ala72, shaping a high-energy thioester bond. The activated NEDD8 is subsequently transferred to a cysteine residue on NEDD8-specific E2s through the transthiolation reaction. Ultimately, under the catalysis of NEDD8 E3 ligase, NEDD8 is transferred from the E2s to the substrate protein, covalently bonding to the lysine (Lys) residue. Deneddylases remove the NEDD8 molecule from the substrate, completing the dynamic and reversible neddylation-deneddylation modification process. Protein neddylation orchestrates protein stability, protein subcellular localization, DNA damage response, transcriptional regulation, protein function, and enzymatic activity, therefore manipulating critical molecular processes in tumors, such as gene expression, signaling transduction, and RNA metabolism. This modulation contributes to the imbalance of cellular homeostasis in tumors. The figure was drawn by authors for this paper.

Neddylation comprises a succession of enzymatic reactions, involving maturation, activation, conjugation, ligation, and deneddylation [16] (Fig. 1). Mechanistically, hydrolases, involving UCH-L3 and NEDP1, proteolytically cleave the C-terminal amino acids of NEDD8 precursor to expose the Gly-Gly motif, resulting in the formation of a 76-amino-acid mature NEDD8 [15, 17]. The mature NEDD8 interacts with the active site cysteine of NAE *via* its Ala72, shaping a high-energy thioester bond. The activated NEDD8 is subsequently transferred to a cysteine residue on NEDD8-specific E2s through the transthiolation reaction. Under the catalysis of NEDD8 E3 ligase, NEDD8 is transferred from the E2s to the substrates, covalently bonding to the K residue [7, 18, 19]. Deneddylases remove the NEDD8 molecule from substrates, completing the dynamic and reversible neddylation-deneddylation process. The substrates of neddylation are categorized into cullins and non-cullin proteins. The cullin (CUL) family consists of CUL1, CUL2, CUL3, CUL4A, CUL4B, CUL5, CUL7, and CUL9 [20]. Non-cullin neddylation substrates mainly include signaling molecules such as TP53, TP73, E2F1, epidermal growth factor receptor (EGFR), transforming growth factor-β type II receptor (TGFβRII), NF-κB essential modulator (NEMO), and Von Hippel-Lindau (VHL) [3].

### Biological features of neddylation in tumors

Neddylation manipulates critical molecular processes in tumors, including gene expression, signaling transduction, and RNA metabolism, by orchestrating protein stability, subcellular localization, and transcriptional regulation (Fig. 1, Table 2). Strikingly, Neddylation exhibits a dual regulatory function in maintaining protein homeostasis within tumors. Typically, neddylation influences protein degradation by improving the activities of Cullin-RING E3 ligases (CRLs). Neddylation also modulates protein function through altering the enzyme activities, binding properties, and protein conformation. Representatively, neddylation of the cullin family enhances the activities of CRLs, which account for approximately 20% of proteasome-mediated protein degradation within the ubiquitin-proteasome system (UPS) [21]. Notably, neddylation governs protein binding properties and conformation in tumors, highlighting its functional diversity. The precise regulation of protein subcellular localization is fundamental for controlling cellular functions, metabolism, and homeostasis. Neddylation modulates tumor initiation and progression through regulating protein nuclear and cytoplasmic localization. Moreover, transcription regulation participates in tumor progression, influencing tumor cell propagation, apoptosis, and metabolism. Neddylation serves as a critical regulatory component in transcriptional regulation, interacting with transcription factors (TFs), nuclear receptors, and multiple signaling pathways. Additionally, Neddylation enables tumor cells to manipulate the DNA damage response (DDR), allowing them to sustain propagation, growth, and survival under internal and external stress. Overall, the neddylation cascade plays versatile roles in tumor cells, administering significant molecular processes that contribute to tumor malignant phenotypes. Therefore, understanding the role of neddylation may help identify effective tumor biomarkers and enable personalized treatment approaches.

**Table 2:** Biological features of neddylation in tumors.

Biological feature	Functional role	Mechanism/Example	Tumor type	Refs
Protein stability	Inhibit ubiquitination and degradation	NEDD8 E3 ligase RAPSYN-mediated neddylation of BCR-ABL at K500, K739, K802, K1025, K1135, K1590, and K1990 debilitates c-CBL-mediated ubiquitination	Leukemia	[79]
		c-CBL-mediated neddylation of TβRII at K556 and K567	Leukemia	[143]
		MDM2-mediated neddylation of HuR at K283, K313, and K326	HCC and colon cancer	[144]
	Facilitate degradation	UBE2M-involved neddylation of LSD1 at K63	Gastric cancer	[23]
		neddylation of SRSF3 at K11	HCC	[145]
Protein function	Alter enzyme activities	UBC12-induced neddylation of CUL1 activates E3 ubiquitin ligase CRL1, facilitating the degradation of p27	Breast cancer	[63]
		UBE2F-mediated neddylation of CUL5 stimulates CRL5, enhancing the ubiquitylation of DIRAS2	Pancreatic cancer	[46]
		CSN6-induced neddylation of CUL1 boosts E3 ligase Fbxw7 autoubiquitination in the SCF complex, therefore stabilizing MYC	Lymphoma	[146]
	Change binding properties	neddylation of PDK1 by E3 ligase Smurf1 at K163 facilitates the recruitment of SETDB1, a methyltransferase for Akt activation	KRAS-mutated CRC	[147]
	Modify protein conformation	UBC12-mediated NEDD8 transfer to E3 ubiquitin ligase TRIM25 at K117 reduces steric hindrance in its RING domain, facilitating the interaction between TRIM25 and its substrates	Paclitaxel-resistant TNBC	[65]
		NEDD8 conjugation of SHP2 at K358 and K364 sustains its autoinhibitory conformation, debilitating its activation	Colon cancer	[58]
Subcellular localization	Promote nuclear localization	HDM2-mediated neddylation of HBV-encoded X protein (HBx) at K91 and K95 stabilizes HBx and ensures its nuclear localization	HBV-related HCC	[60]
		PTEN undergoes neddylation mediated by XIAP ligase at K197 and K402, facilitating PTEN nuclear import, thereby stimulating PI3K/Akt pathway	Breast cancer	[49]
	Enhance cytoplasmic localization	Mdm2-mediated neddylation of TAp73 induces its redistribution from the nucleus to the cytoplasm	Pan-cancer	[148]
Transcriptional regulation	Regulate the expression and activity of transcription factors	neddylation of cell-cycle-regulating transcription factor E2F1 at K14 reduces its stability and transcriptional activity	Pan-cancer	[149]
		neddylation of IκBα improves NF-κB transcriptional activity and the transactivation of CCL2 and CXCL6	Lung cancer	[150, 151]
	Degrade nuclear receptors	neddylation-activated Cullin subunits of the SCF complex promote the degradation of the nuclear receptor ERRβ, leading to reduced transcription of p21 and E-cadherin	Breast cancer	[152]
	Stabilize transcriptional co-regulators	deneddylation of CUL1 and dysfunction of E3 ligase SCF stabilize transcriptional co-regulators YAP/TAZ	HCC	[62]
	Induce signaling transduction	UBE2F-mediated neddylation of CUL5 activates CRL5 complex, inhibiting L-15R signaling	Pan-cancer	[153]
DNA damage response	Impair p53-dependent DNA repair	UBC12-mediated neddylation of E3 ubiquitin-protein ligase MDM2 facilitates p53 degradation	Pan-cancer	[154]
		DYRK2-NAE1 complex-induced neddylation activates CRL-mediated proteasome degradation, leading to destabilization and inactivation of p53 and DNA double-strand breaks	Pan-cancer	[155]

### Neddylation-maintained cancer hallmarks

**Fig. 2:**
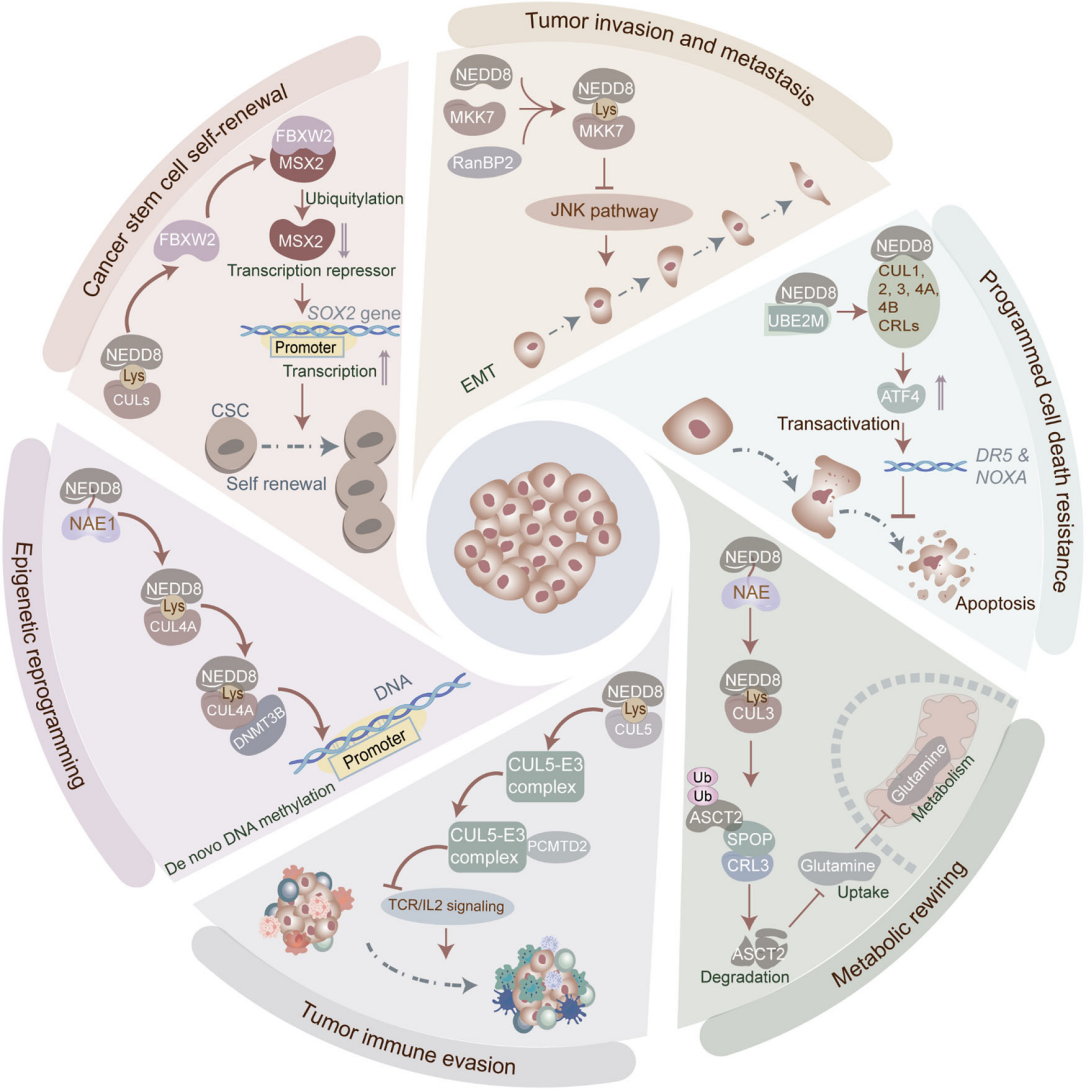
Neddylation-maintained cancer hallmarks. The neddylation cascade is widely recognized as a critical factor in tumor onset, progression, and therapeutic response. Neddylation exerts an important effect on sustaining tumor malignant phenotypes, involving invasion, metastasis, cancer stem cell self-renewal, programmed cell death resistance, metabolic rewiring, immune evasion, and epigenetic reprogramming. In breast cancer, RanBP2-mediated neddylation of MKK7 constrains its basal kinase activity and inhibits basal JNK phosphorylation, thus facilitating proliferation and EMT. In ESCC, UBC12-involved neddylation of CUL1, CUL2, CUL3, CUL4A, and CUL4B induces the activation of CRLs, leading to the depletion of ATF4 and transrepression of DR5, which inhibits extrinsic apoptosis. ATF4 reduction also decreases the expression of apoptotic protein NOXA, inactivating intrinsic apoptosis. In breast cancer, the CRL3-SPOP E3 ligase, activated by neddylation, mediates the ubiquitination and K48-linked degradation of ASCT2 by interacting with the SPOP consensus motif “GTSSS,” thereby reducing cellular glutamine uptake and glutamate production. Neddylation-induced activation of the CUL5-E3 ligase complex, interacting with SOCS-box-containing protein PCMTD2, impedes TCR/IL2 signaling and reduces CD8^+^ T cell cytotoxicity in tumors. CUL4A preferentially binds to DNMT3b *via* its C terminus in a neddylation-dependent manner, therefore promoting DNMT3b-mediated DNA methylation in cancer cells. Neddylation-activated CRL E3 ligase FBXW2 mediates the ubiquitination and degradation of MSX2, a transcription repressor of SOX2, therefore upregulating SOX2 expression and boosting stem cell properties in breast cancer. The figure was drawn by authors for this paper.

**Fig. 3:**
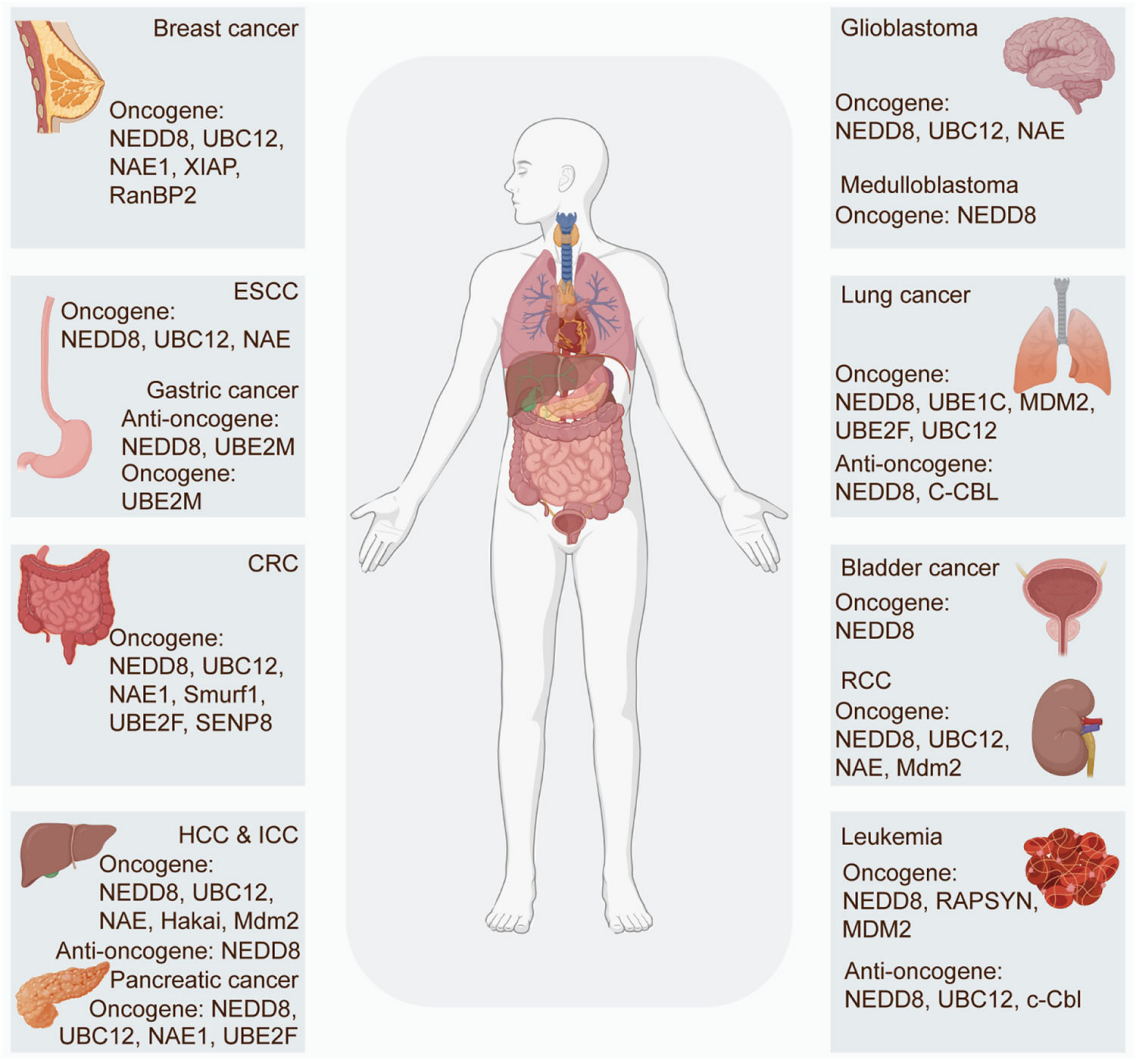
Role of neddylation molecules in tumors. Mounting evidence robustly links neddylation to tumor development. For instance, UBE2F overexpression facilitates CUL5 neddylation and triggers CRL5 to ubiquitinate NOXA at K11, which supports lung cancer cell survival. However, UBE2M-mediated neddylation of LSD1 at K63 enhances its ubiquitination and degradation, reducing the stemness and chemoresistance in GC. Thus, it can be concluded that neddylation has shown both tumor-suppressive and tumor-promoting effects in certain contexts, depending on the cancer type and the cellular microenvironment. Created with BioRender.com.

Neddylation exerts an important effect on sustaining tumor malignant phenotypes (Fig. 2, Table 3). However, it presents tumor-suppressive effects in certain contexts, depending on the cancer type and the tumor microenvironment (TME) (Fig. 3). Specifically, UBE2F, in conjunction with E3 ligase SAG, neddylates RHEB at K169, boosting its lysosome localization and GTP-binding affinity, thereby exacerbating HCC tumorigenesis [22]. Whereas, UBE2M-mediated neddylation of LSD1 at K63 enhances its ubiquitination and degradation, reducing the stemness and chemoresistance in GC [23]. These observations underscore the intricate role of neddylation in tumorigenicity, with accumulating evidence robustly establishing its role in tumor development. Herein, we investigate the underlying mechanisms by which neddylation sustains cancer hallmarks, enhancing the understanding of cancer biology and providing novel avenues for therapeutic intervention.

**Table 3:** The function and mechanism of neddylation molecules in cancers.

Cancer type	NEDD8 enzyme	Substrate	Neddylation sites	Function and mechanism	Refs
Bladder cancer	NEDD8	Unknown	Unknown	promote proliferation, migration, and invasion of tumor cells	[156]
Breast cancer	NEDD8, UBC12	TRIM25	K117	decrease tumor sensitivity to PTX via reducing steric hindrance of TRIM25	[65]
	UBC12	CUL1	WHB domain	facilitate tumor progression via neddylation-mediated p27 degradation	[63, 157]
	NEDD8, NAE1	HER2	Unknown	promote tumor progression via inhibiting HER2 degradation	[158]
	NEDD8, XIAP	PTEN	K197, K402	promote tumor progression via regulating PTEN nuclear import	[49]
	NEDD8, RanBP2	MKK7	Unknown	facilitate tumor cell proliferation and EMT via inhibiting basal JNK phosphorylation	[28]
CRC	NAE1, Ubc12, Smurf1	PDK1	K163	promote CRC tumorigenesis via PI3K-Akt pathway	[147, 159]
	UBE2F	CUL5	WHB domain	inhibit apoptosis and promote etoposide resistance of CRC cells via inducing NOXA ubiquitination and degradation	[36, 160]
	NEDD8	CUL4A, CUL4B	WHB domain	activate the DCAF13-CRL4 ubiquitin ligase complex to induce the resistance of topoisomerase I inhibitors	[97]
	SENP8	SHP2	K358, K364	SHP2 activates and recruits SIRPαto suppress macrophage phagocytosis via SENP8-mediated deneddylation	[58]
	Smurf1	RRP9	K221	promote tumor cell proliferation via pre-rRNA processing	[161]
ESCC	NEDD8	CULs	WHB domain	promote malignant phenotypes via reducing CRL substrates, including p21, p27, and Wee1	[162]
	NEDD8	CUL1	WHB domain	inhibit the accumulation of IκBα and protective autophagy via NF-κB/Catalase/ATF3 pathway	[41]
	NAE, UBC12	CULs	WHB domain	inhibit apoptosis through ATF4-CHOP-DR5 axis	[34]
GC	NEDD8, UBE2M	LSD1	K63	decrease GC stemness via promoting LSD1 ubiquitination and degradation	[23]
	UBE2M	CUL3, CUL5	WHB domain	promote tumor growth and inhibit apoptosis via decreasing the accumulation of Nrf2 and NOXA	[123]
Glioblastoma	NEDD8, NAE, UBC12	CUL1	WHB domain	inhibit senescence or apoptosis via decreasing p21 and p27	[99]
	NEDD8	CUL1	WHB domain	decrease c-MYC expression via increasing CUL1-FBXW7 E3 ligase activity	[57]
Medulloblastoma	NEDD8	p21	Unknown	promote medulloblastoma cell proliferation via increasing cIAP1-mediated p21 degradation	[163]
HCC	UBE2F	RHEB	K169	aggravate liver tumorigenesis via enhancing mTORC1activity	[22]
	NEDD8, NAE	SRSF3	K117	contribute to progressive liver disease via inducing SRSF3 proteasome-mediated degradation	[145]
	Hakai	Ajuba	Unknown	promote hepatocellular carcinoma cell growth via inducing β-catenin translocation and enhancing YAP signaling	[164]
	NEDD8, Mdm2	HuR	K283, K313, K326	promote malignant transformation via ensuring HuR nuclear localization and inhibiting HuR degradation	[144]
	NEDD8	CUL1	WHB domain	promote the YAP/TAZ pathway via impairing SCFβ-TrCP E3 ligase activity	[62]
HNSCC	NAE1, UBC12	CULs	WHB domain	inhibit apoptosis via c-Myc-Noxa axis	[165]
	NEDD8	CUL4	WHB domain	promote cancer progression via activating the mTOR signaling pathway	[29]
ICC	NEDD8, NAE1	CULs	WHB domain	promote tumorigenesis via reducing both cullin RING ligase and NEDD8 substrates	[166]
	NAE, UBC12	CULs	WHB domain	promote malignant phenotypes via decreasing tumor-suppressive CRL substrates	[167]
Leukemia	NEDD8	HDAC1	Unknown	reduce doxorubicin resistance by Nedd8-mediated neddylation and ubiquitination	[69]
	NEDD8	RARα	K227, K360	accelerate leukemogenesis via destroying the formation of PML nuclear bodies	[168]
	NEDD8, UBC12, c-Cbl	TβRII	K556, K567	suppress leukemia formation by amplifying the antiproliferative signals from TGF-β receptors	[143]
	RAPSYN	BCR-ABL	K500, K739, K802, K1025, K1135, K1590, K1990	facilitate leukemia progression by boosting the protein stability of BCR-ABL	[79]
	NEDD8, MDM2	p53	K120	reduce the viability of leukemic cells via diminishing p53 tumor suppressor activity	[169]
Lung cancer	NEDD8, C-CBL	c-Src	Unknown	blunt cell migration via inhibiting the PI3K-AKT pathway	[170]
	MDM2	RPS27L, RPS27	Unknown	confer the survival of cancer cells via stabilizing RPS27L and RPS27	[171]
	UBE2F	CUL5	WHB domain	facilitate the survival of cancer cells via CRL5-mediated degradation of NOXA	[35, 64]
	NEDD8, UBE1C	p53	Unknown	promote migration, invasion, and proliferation of lung cancer cells by reducing p53 transcriptional activity	[172]
	UBC12	CUL1, 2, 3, 4 A, 4B	WHB domain	facilitate the growth and metastasis of lung cancer by downregulating CRLs substrates	[120]
Melanoma	NAE1, UBA3, UBC12	CUL1	WHB domain	promote hepatic metastasis by increasing cancer stem-like cells and angiogenesis	[26]
Pancreatic cancer	NEDD8, NAE1, Ubc12	CUL4B	WHB domain	promote tumor progression via activating CUL4B-DCAF7 axis	[82]
	UBE2F	CUL5	WHB domain	facilitate pancreatic tumorigenesis via activating UBE2F-CRL5ASB11 axis	[46]
Prostate cancer	DCUN1D1	CUL1, 3, 4A, 4B, 5	WHB domain	promote tumor growth and inhibit apoptosis via WNT Pathway	[173]
RCC	NAE, UBC12	CUL1	WHB domain	promote proliferation and migration via reducing the accumulation of p21, p27, and Wee1	[174]
	Mdm2	VHL	K159	promote angiogenesis through the regulation of the VHL-p53 complex formation	[25]

### Tumor invasion and metastasis

The invasion and spread of tumor cells influence the treatment strategies and patient prognosis. Remarkably, neddylation participates in tumor invasion and metastasis *via* regulating tumor angiogenesis and epithelial-to-mesenchymal transition (EMT). Targeting neddylation hinders the metastatic process, involving intravascular survival, extravasation, and formation of metastatic clusters [24]. Tumor angiogenesis not only supplies nutrients to tumor cells but also facilitates their dissemination through the bloodstream. Notably, neddylation manipulates tumor angiogenesis. Mdm2-mediated neddylation of VHL tumor suppressor protein at K159 disrupts VHL-p53 complex formation, fostering tumor angiogenesis [25]. Moreover, NAE1-mediated neddylation of CUL1 upregulates vascular endothelial growth factor C (VEGF-C) by augmenting NF-κB transcriptional activity, thereby facilitating tumor angiogenesis and uveal melanoma hepatic metastasis [26]. Furthermore, cullin neddylation-mediated activation of CRL reduces RhoA accumulation and increases the angiogenic activity of vascular endothelial cells, contributing to tumor angiogenesis [27]. These findings demonstrate that neddylation acts as an accomplice in tumor angiogenesis.

EMT enables tumor cells to transition from an epithelial to a mesenchymal phenotype, enhancing their ability to invade and migrate. Neddylation regulates EMT in tumors. RanBP2-mediated neddylation of MKK7 constrains its basal kinase activity and inhibits basal JNK phosphorylation, thus facilitating proliferation and EMT in BC cells [28]. Moreover, neddylation of tuberous sclerosis complex 2 (TSC2) inactivates the mTOR pathway, enhancing migration, invasion, and EMT in head and neck squamous cell carcinoma (HNSCC) [29]. However, neddylation also impedes the EMT process. Concretely, neddylation inactivates hypoxia-inducible factor 1α (HIF-1α) *via* PI3K-Akt signaling, downregulating zinc finger E-box binding homeobox1 (ZEB1) and inhibiting EMT in cancer cells [30]. Interestingly, neddylation governs tumor cell migration in a manner relying on the p53 status. Specifically, in the presence of wild-type p53, neddylation blockage increases p53 transcriptional activity and enhances p21 and MDM2 expression, ultimately leading to the proteasomal degradation of Slug and impeding EMT-involved tumor cell migration. Conversely, in cells with mutant p53, neddylation inhibition facilitates tumor cell migration by stimulating the PI3K/Akt/mTOR/Slug pathway [31].

Taken together, these findings highlight the dual function of the neddylation cascade in tumor invasion and metastasis, complicating the regulatory mechanisms that control the invasive-metastatic process through neddylation and raising concerns about the use of MLN4924 as an anti-tumor therapeutic agent.

### Programmed cell death resistance

Programmed cell death (PCD) sustains physiological homeostasis by eliminating abnormal or harmful cells. Tumor cells resist PCD, endowing them with prolonged survival [32]. Notably, neddylation dysregulates the PCD process in tumors, affecting apoptosis, autophagy, and ferroptosis. Apoptosis inhibition is critical for tumor cell survival. Neddylation governs the apoptotic pathways, manipulating the tumor cell fate. In ESCC, UBC12-involved neddylation of CUL1, CUL2, CUL3, CUL4A, and CUL4B activates CRLs, leading to the depletion of activating transcription factor 4 (ATF4) and transrepression of death receptor 5 (DR5), which inhibits extrinsic apoptosis. ATF4 reduction also downregulates apoptotic protein NOXA, inactivating intrinsic apoptosis [33]. Conversely, blocking neddylation stabilizes ATF4, leading to the transactivation of TF CHOP. This upregulates DR5 and caspase-8, triggering extrinsic apoptosis [34]. Additionally, UBE2F-mediated neddylation activates CRL5, enhancing the ubiquitination and degradation of NOXA, thereby facilitating apoptosis resistance in lung cancer and CRC [35, 36]. Autophagy, which degrades intracellular organelles, proteins, and macromolecules, maintains cellular homeostasis. The neddylation cascade participates in autophagy dysregulation during tumorigenesis. Representatively, neddylation hampers autophagy through activating mTOR signaling in tumors. For instance, UBE2F-induced neddylation of RHEB at K169 attenuates autophagy in liver cancer by fortifying mTORC1 activity, thereby promoting tumor growth and survival [22]. Similarly, CUL-RING E3 ligases-induced DEPTOR depletion prevents autophagy in an MTOR-dependent manner, worsening tumorigenesis [37, 38]. Additionally, neddylation manipulates autophagy in tumors through HIF1-REDD1-TSC1-mTORC1-DEPTOR axis [39], PI3K/AKT/mTOR signaling [40], and NF-κB-catalase-ATF3 axis [41]. These findings highlight that neddylation dampens autophagy in tumors, thereby fostering the malignant phenotypes. Neddylation also governs ferroptosis in tumors. Mechanistically, neddylation E3 CRL-3 decreases cystine availability by destabilizing the cystine/glutamate antiporter SLC7A11, leading to ferroptosis resistance in BC [42]. Overall, PCD resistance is tightly linked to tumor deterioration and therapeutic resistance. Accordingly, elucidating the mechanisms by which neddylation mediates PCD evasion in tumor cells could provide novel therapeutic avenues and diagnostic approaches in oncology.

### Cancer stem cell self-renewal and maintenance

Cancer stem cells (CSCs) are a subpopulation of tumor cells with extraordinary abilities to self-renew, differentiate, and maintain tumor malignancy. Strikingly, the overall level of neddylation in tumors significantly influences CSC self-renewal and maintenance. Slug maintains CSC traits by enhancing its stability in a neddylation-dependent manner [26]. Moreover, neddylation controls the expression of SRY-box transcription factor 2 (SOX2), crucial for CSC self-renewal. Mechanistically, neddylation-activated CRL E3 ligase FBXW2 mediates the ubiquitination and degradation of MSX2, a transcription repressor of SOX2, therefore upregulating SOX2 expression and boosting stem cell properties in BC [43]. However, neddylation also attenuates tumor stemness. Specifically, UBE2M-mediated neddylation of LSD1 at K63 enhances its ubiquitination and degradation, thereby reducing GC cell stemness [23]. Similarly, c-MYC, an oncoprotein that promotes CSC self-renewal, is ubiquitylated and degraded by SCF E3 ubiquitin ligases, including SCF^FBXW7^ and SCF^SKP2^, hindering tumor sphere formation, stem cell proliferation, and differentiation [44]. CSC self-renewal and maintenance are critical for tumor growth, metastasis, therapy resistance, and recurrence. Targeting CSCs offers potential treatment options for refractory and recurrent cancers. While neddylation inhibition effectively targets tumor heterogeneity, its dual role in CSC self-renewal/maintenance complicates therapeutic application, posing potential risks and challenges.

### Epigenetic reprogramming

Epigenetic reprogramming regulates gene expression by altering epigenetic markers without altering the DNA sequence, playing a key role in tumor initiation, progression, metastasis, and therapeutic resistance. In tumors, neddylation serves as an epigenetic modification that coordinates other epigenetic changes, including DNA methylation, transcriptional regulation, and PTMs. Neddylation influences the activity of proteins or enzymes involved in DNA methylation, thus indirectly altering the DNA methylation pattern. Specifically, CUL4A preferentially binds to DNA methyltransferase 3b (DNMT3b) *via* its C terminus in a neddylation-dependent manner, therefore promoting DNMT3b-mediated DNA methylation in cancer cells [45]. Moreover, neddylation regulates other PTM processes in tumors. Characteristically, neddylation modification enables the CRL complex to recognize target proteins and enhances their degradation *via* the ubiquitin-lysosome pathway. UBE2F, in association with the neddylation E3 ligase RBX2/SAG, triggers the neddylation of CUL5, thus activating E3 CRL5. Subsequently, CRL5 mediates the degradation of DIRAS2 through K11-linked polyubiquitylation in KRAS mutant pancreatic cancer cells [46]. Similarly, CUL5 neddylation enhances CRL5 activity through the UBE2F/SAG/CUL5 complex, thus ubiquitylating NOXA at K11 in the proteasomal degradation pathway in lung cancer [35]. Neddylation also manipulates transcription by modulating the expression and activity of TFs, nuclear receptors, and transcriptional co-regulators in tumors. Neddylation-driven epigenetic reprogramming promotes tumor cell heterogeneity and clonal evolution. Therapeutic targeting of neddylation may offer a multidimensional strategy to eliminate malignant cells through multiple mechanisms.

### Metabolic rewiring

Metabolic rewiring enables tumor cells to modify their metabolic pathways to adapt to the hypoxic, nutrient-deprived, acidic TME, supporting proliferation, immune evasion, and therapy resistance. Remarkably, neddylation supervises metabolic rewiring in tumors, influencing aerobic glycolysis, glutamine metabolism, and fatty acid synthesis. Aerobic glycolysis, or the Warburg effect, is a hallmark metabolic adaptation in tumors, which is regulated by neddylation. Specifically, glucose detaches CRL4 from the deneddylase CSN, contributing to the assembly of the CRL4^COP1^ E3 complex. This complex facilitates the degradation of wild-type p53, therefore enhancing the Warburg effect and driving mammary tumorigenesis [47]. Moreover, neddylation governs amino acid metabolism in tumors. The CRL3-SPOP E3 ligase, activated by neddylation, mediates the ubiquitination and K48-linked degradation of ASCT2 by interacting with the SPOP consensus motif “GTSSS,” thereby reducing cellular glutamine uptake and glutamate production in BC [48]. Furthermore, neddylation regulates fatty acid metabolism in cancers. Specifically, XIAP-mediated neddylation of PTEN at K197 and K402 reinforces its accumulation in the nucleus, potentiating FASN dephosphorylation and attenuating FASN ubiquitination, which accelerates de novo fatty acid synthesis in BC [49]. Collectively, these findings underscore the essential role of neddylation in controlling metabolic rewiring in tumors. Hence, targeting neddylation may enhance therapeutic outcomes, surmount tumor immunosuppression, and ameliorate the TME by altering the metabolic profile of tumor cells, providing a potential strategy for personalized cancer treatment.

### Tumor immune evasion

The immune system recognizes and eliminates abnormal or harmful cells. Tumor cells evade immune surveillance through multiple mechanisms, including the neddylation cascade, thereby expediting tumor growth and metastasis. Strikingly, the Warburg effect exacerbates immune evasion in tumors [50, 51]. Neddylation dysregulates T-cell cytotoxicity within tumors. For instance, neddylation-induced activation of the CUL5-E3 ligase complex, interacting with SOCS-box-containing protein PCMTD2, impedes TCR/IL2 signaling and reduces CD8^+^ T cell cytotoxicity in tumors [52]. Therefore, pharmacologic blockage of neddylation effectively revitalizes T-cell response in malignancies [53, 54]. Neddylation also governs the cytotoxic activity of Natural Killer (NK) cells in tumors. Neddylation upregulates MICA and MICB, the ligands of NK cell-activating receptor NKG2D, by modulating MICA promoter activity and MICB subcellular localization, contributing to NK cell degranulation [55]. Furthermore, neddylation aggravates the infiltration of tumor immunosuppressive cells. Overexpression of UBA3 facilitates the infiltration of tumor-associated macrophages (TAMs), plasmacytoid dendritic cells (pDCs), Th2 cells, and T-regulatory cells (Tregs) by decreasing phosphorylated nuclear factor of kappa light polypeptide gene enhancer in B-cells inhibitor (p-IκBα) and improving the gene expression of tumor cell-derived cytokines in lung adenocarcinoma [56]. Contrariwise, neddylation counteracts tumor immune evasion by downregulating the expression of immune checkpoint molecules. For instance, neddylation augments the activity of Cullin1-F-box and WD repeat domain-containing 7 E3 ligase, which destabilizes c-MYC protein, decreasing PD-L1 expression and improving T cell killing in glioblastoma [57]. Intriguingly, deneddylation also contributes to tumor immune evasion by restraining macrophage phagocytosis in colon cancer. Mechanistically, neddylation of Src homology region 2-containing protein tyrosine phosphatase 2 (SHP2) at K358 and K364 disturbs its activity and sustains its autoinhibited conformation. Deneddylation of SHP2 by sentrin-specific protease 8 (SENP8) inactivates αMβ2 integrin, ultimately impairing macrophage phagocytosis in colon cancer [58]. Neddylation exerts dual immunomodulatory effects in tumors, with outcomes determined by its regulation of tumor cells, immune components, and the TME. Understanding the mechanisms underlying tumor immune evasion provides a foundation for tumor immunotherapy.

### Cellular stresses triggering neddylation in tumors

**Fig. 4:**
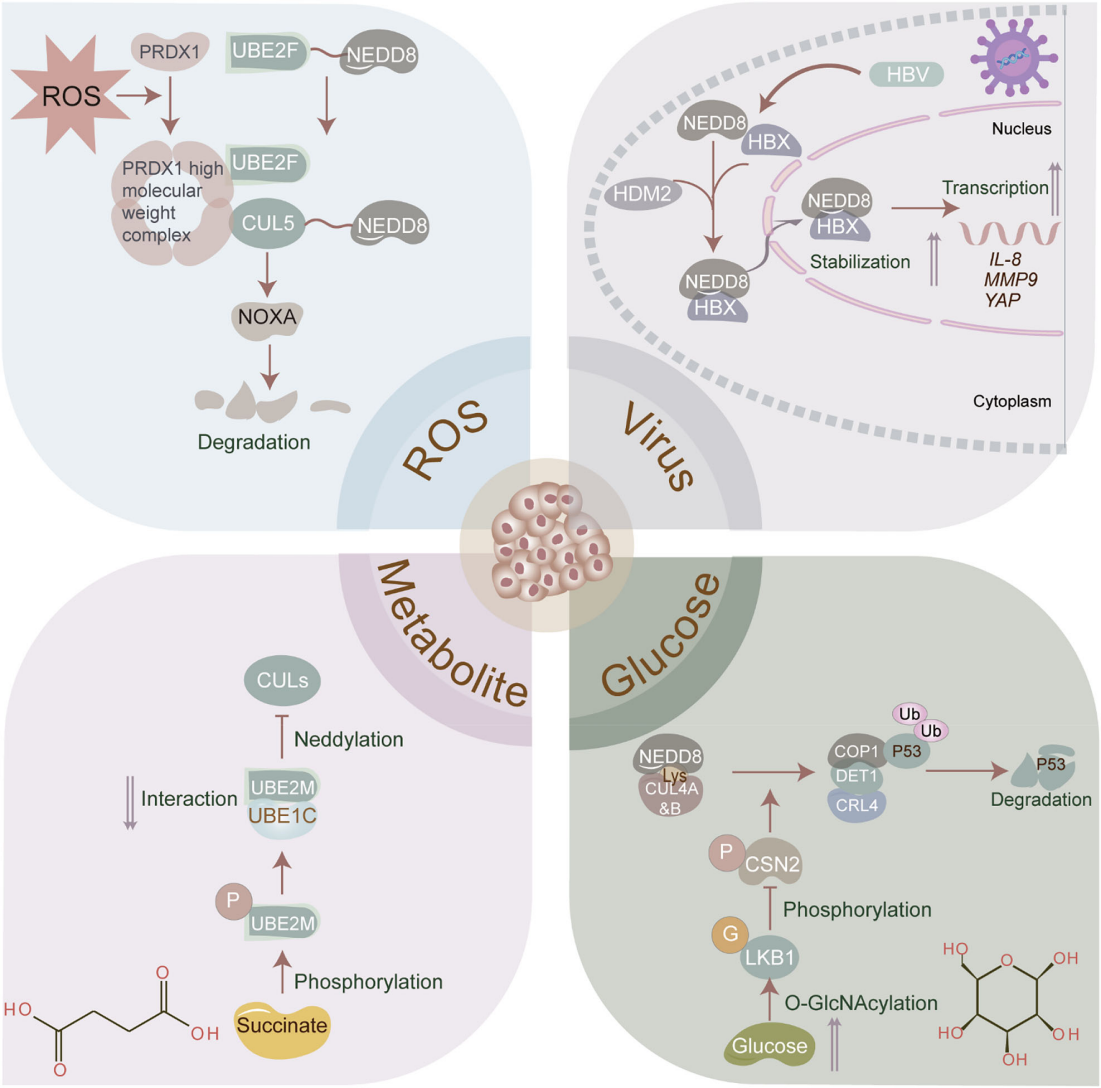
Cellular stresses triggering neddylation in tumors. The neddylation cascade is vital for maintaining cellular homeostasis and is triggered by various forms of cellular stress. Neddylation is essential for the cellular response to DNA damage in tumors. In reaction to chemotherapy-induced DNA damage, ROS induces UBE2F-mediated neddylation of CUL5, facilitating NOXA ubiquitination and degradation, which confers chemoresistance to CRC cells. Viruses can promote cancer development, in part, by hijacking the neddylation pathway. In Hepatitis B virus-associated HCC, neddylation of HBx facilitates its nuclear localization, transcriptional activity, and stabilization, thereby activating the transcription of IL-8, MMP9, and YAP, which accelerates tumor growth. High glucose conditions can stimulate tumor initiation and progression by regulating the neddylation cascade. High glucose dissociates CRL4 from the deneddylase CSN, assembling the CRL4^COP1^ E3 complex. This, in turn, leads to the degradation of wild-type p53, accelerating mammary tumorigenesis. Metabolites also modulate neddylation by influencing enzyme activity, thereby linking cellular metabolism with protein function in tumors. succinate induces the phosphorylation of E2 enzyme UBC12 at the serine-6 site, impairing cullin neddylation by undermining UBC12/UBE1C interaction, ultimately stabilizing oncoproteins in AML. The figure was drawn by authors for this paper.

Neddylation, maintaining cellular homeostasis, is triggered by various forms of cellular stress. However, the specific stress that induces neddylation and the mechanisms through which it responds remain incompletely understood. This section explores the different stimuli and the mechanisms that induce neddylation in tumor cells (Fig. 4). Neddylation is essential for the cellular response to DNA damage in tumors. In reaction to chemotherapy-induced DNA damage, reactive oxygen species (ROS) induces UBE2F-mediated neddylation of CUL5, facilitating NOXA ubiquitination and degradation, which confers chemoresistance to CRC cells [36]. Furthermore, viruses promote cancer development, in part, by hijacking the neddylation pathway. Specifically, Kaposi’s sarcoma-associated herpesvirus (KSHV), the primary cause of Kaposi’s sarcoma, activates NF-κB signaling by recruiting the neddylated CUL1-activated CRL1/βTrCP E3 ligase to degrade IκBα. More importantly, neddylation also enhances KSHV genome replication during the reactivation of the lytic cycle [59]. In Hepatitis B virus-associated HCC, neddylation of HBx facilitates its nuclear localization, transcriptional activity, and stabilization, thereby activating the transcription of IL-8, MMP9, and YAP, which accelerates tumor growth [60]. High glucose conditions, a known oncogenic factor, stimulate tumor initiation and progression by regulating the neddylation cascade. Under hyperglycemic conditions, PTEN neddylation at K197 and K402 facilitates its nuclear import without altering protein stability, thus exacerbating BC progression [49]. Likewise, high glucose dissociates CRL4 from the deneddylase CSN, assembling the CRL4^COP1^ E3 complex. This, in turn, leads to the degradation of wild-type p53, accelerating mammary tumorigenesis [47]. Strikingly, metabolites also modulate neddylation by influencing enzyme activity, thereby linking cellular metabolism with protein function in tumors. Concretely, succinate induces the phosphorylation of UBC12 at the serine-6 site, impairing cullin neddylation by undermining UBC12/UBE1C interaction, ultimately stabilizing oncoproteins in AML [61]. Succinate accumulation also enhances the deneddylation of cullin1, impairing the E3 ubiquitin ligase SCF β-TrCP complex, which stabilizes and activates YAP/TAZ in HCC [62]. Additionally, 1-methyl-nicotinamide (1-MNA), a metabolite of nicotinamide N-methyltransferase (NNMT), is critical for CRL1 activation. Mechanistically, NNMT/1-MNA directly binds to UBC12 and impedes UBC12 lysosome degradation, subsequently triggering cullin-1 neddylation and leading to p27 protein decay in BC [63].

These findings indicate that cellular stress within the TME activates adaptive responses that contribute to tumor cell survival and progression. Addressing cellular stress, such as oxidative stress, metabolic pressure, and DNA damage, can help maintain cellular homeostasis, preventing tumor initiation, progression, and resistance. Targeting the cellular stress triggering neddylation in tumors, therefore, holds potential for reducing the adaptability of tumor cells and enhancing treatment efficacy.

### Neddylation-involved therapeutic resistance in tumors

**Fig. 5:**
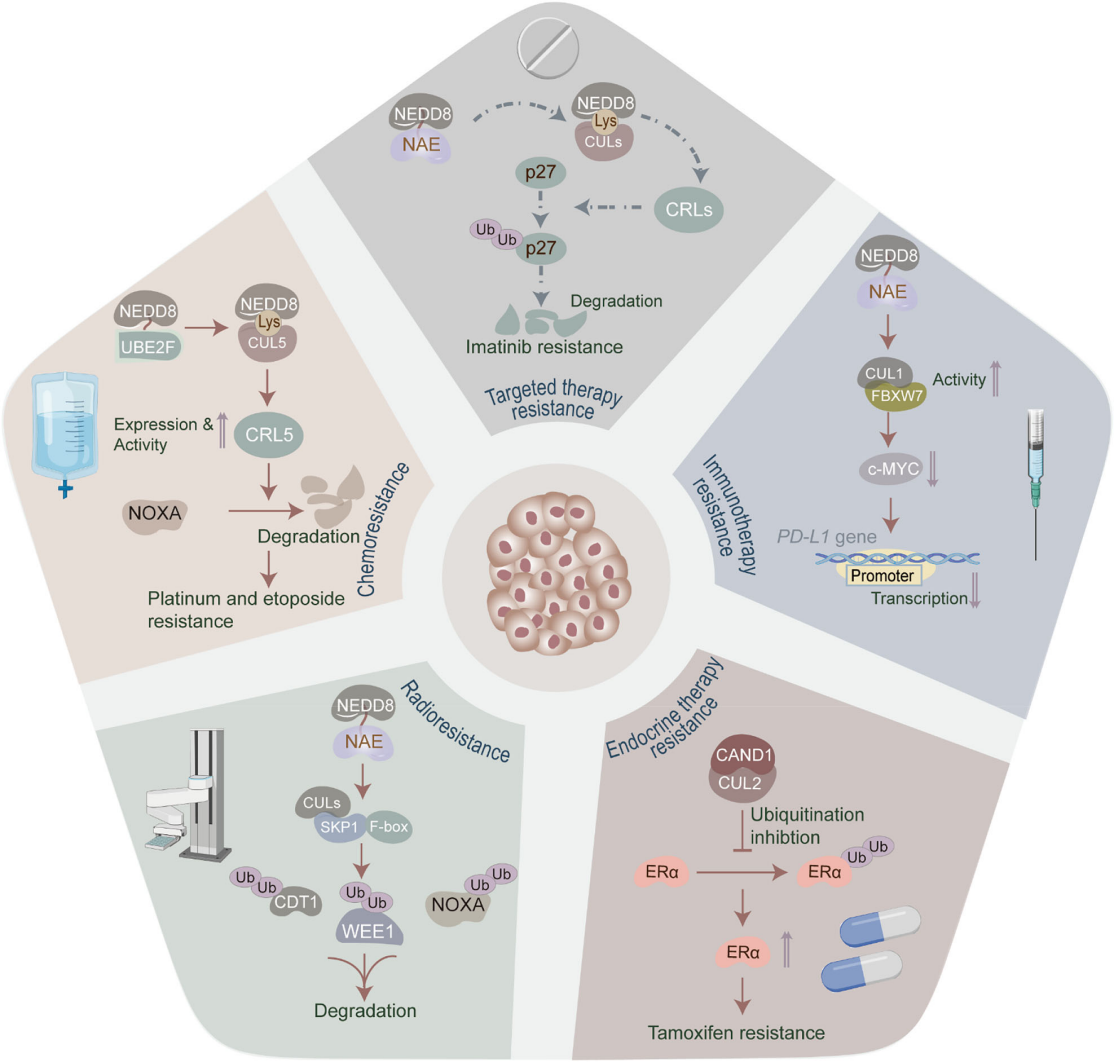
Neddylation-involved therapeutic resistance in tumors. Tumor resistance poses a tremendous challenge in oncology. Neddylation has been implicated in tumor therapeutic resistance. Upregulation of NEDD8-conjugating enzyme UBE2F enhances the neddylation levels and activity of CUL5, thereby reinforcing CRL5-mediated degradation of NOXA and leading to platinum and etoposide resistance in NSCLC and CRC. Neddylation reduces radiation-induced DNA damage, aneuploidy, G(2)/M phase cell-cycle arrest, and apoptosis through the degradation of SCF substrates, such as CDT1, WEE1, and NOXA. In CML, upregulated NAE1-mediated neddylation of cullins depletes CRL substrate p27 in the nucleus, thereby retaining leukemia stem cells and conferring imatinib resistance. In breast cancer, CAND1, a regulator of cullin-RING E3 ubiquitin ligases, prevents estrogen receptor α (ERα) from CUL2-mediated proteasomal degradation. This preservation of ERα stability with AKTIP loss contributes to the development of tamoxifen resistance. In glioblastoma, neddylation can enhance the activity of the Cullin1-FBXW7 E3 ligase, therefore reducing the accumulation of c-MYC proteins, which transcriptionally decreases *PD-L1* mRNA levels and reduces the efficacy of immune checkpoint blockade. Created with BioRender.com.

Tumor resistance poses a tremendous challenge in oncology, impacting treatment efficacy, patient survival, and prognosis. Resistant tumor cells withstand various therapeutic strategies, including chemotherapy, radiotherapy, targeted therapy, and immunotherapy, raising the risk of recurrence. Remarkably, neddylation contributes to tumor therapeutic resistance (Fig. 5).

### Neddylation-mediated chemoresistance

Chemotherapy, a conventional anti-tumor therapeutic modality, faces the issue of chemoresistance, which contributes to tumor progression and relapse, ultimately affecting patient prognosis and survival. In tumor cells, an overactive neddylation cascade is associated with the development of wide-ranging chemoresistance, desensitizing to platinum, etoposide, and paclitaxel. Specifically, UBE2F upregulation enhances the neddylation levels and CUL5 activity, thereby reinforcing CRL5-mediated degradation of NOXA and leading to platinum and etoposide resistance in non-small cell lung cancer (NSCLC) and CRC [36, 64]. Furthermore, UBC12-mediated TRIM25 neddylation at K117 diminishes steric hindrance in its RING domain, enhancing its ability to bind ubiquitylated substrates. This, in turn, allows TRIM25 to facilitate the nuclear translocation of transcription factor EB (TFEB) and the transcription of autophagy-related genes by increasing the K63-polyubiquitination of TFEB, ultimately reducing the sensitivity of TNBC cells to paclitaxel [65]. Intriguingly, neddylation inhibition presents a promising strategy to overcome cisplatin and paclitaxel resistance in tumors [65**–**68]. Notably, disturbing cullin neddylation by attenuating E2 enzyme UBC12 activity accumulates downstream CRLs, thus inducing chemoresistance in AML [61], indicating that neddylation manipulates chemotherapy sensitivity. Likewise, neddylation blockage hinders the ubiquitination of histone deacetylase 1 (HDAC1) in AML, contributing to its significant upregulation and doxorubicin resistance [69].

### Neddylation-mediated radioresistance

Radiotherapy prevents tumor deterioration by utilizing high-energy radiation to damage the DNA of cancer cells, disrupting their ability to divide and replicate, ultimately leading to tumor cell death. Whereas, protein neddylation confers radioresistance to tumor cells. Of note, overexpression of NEDD8 has been deemed a contributor to radioresistance [70], and neddylation blockage boosts radiation-induced DNA damage, aneuploidy, G(2)/M phase cell-cycle arrest, and apoptosis through the accumulation of SCF substrates, such as CDT1, WEE1, and NOXA [71]. Similarly, neddylation inhibition elevates the radiosensitivity of HNSCC by CRL4^CDT2^-mediated ubiquitination of CDT1, SET8, and p21 [72]. Moreover, CUL1 neddylation has been shown to diminish the radiosensitivity of CRC [73]. UBE2F facilitates CUL5 neddylation and activation, which ubiquitinates and degrades pro-caspase 3, IκBα, and NOXA, thus inducing adaptive radioresistance in lung cancer [74, 75]. Interestingly, cell division cycle (CDC6), a critical molecule assisting tumor cells in resisting radiation, is degraded *via* the CUL1 neddylation-mediated ubiquitin-proteasome pathway, indicating that neddylation also contributes to radiosensitization [76]. Likewise, overexpression of CUL2, functioning as a scaffold for CRL2^VHL^ E3 ligase, negatively correlates with HIF-1α, VEGF-A, Cyclin B1, and EGFR levels, enhancing radiosensitivity in glioblastoma [77]. Additionally, neddylation of p53 enhances its transcriptional activity, therefore increasing the sensitivity to ionizing radiation in HNSCC [78].

### Neddylation-mediated targeted therapy resistance

Tumor-targeted therapy is a highly selective cancer treatment method that impedes tumor growth and metastasis by targeting specific molecules or signaling pathways in cancer cells while minimizing damage to non-tumor cells. Neddylation contributes to targeted therapy resistance, involving tyrosine kinase inhibitor (TKI) resistance and mTOR inhibitor resistance. Concretely, neddylation of BCR-ABL at K500, K739, K802, K1025, K1135, K1590, and K1990, mediated by the NEDD8 E3 ligase RAPSYN, impairs c-CBL-driven ubiquitination, enhancing BCR-ABL protein stability and promoting TKI resistance in Philadelphia chromosome-positive (Ph + ) leukemia [79]. Furthermore, NAE1-mediated neddylation of cullins depletes CRL substrate p27^kip1^ in the nucleus, thereby retaining leukemia stem cells and conferring imatinib resistance in chronic myeloid leukemia (CML) [80]. In contrast, blocking neddylation sensitizes CML and Ph+ acute lymphoblastic leukemia (ALL) to TKIs targeting the ABL kinase [81]. Moreover, neddylation induces mTOR inhibitor resistance in pancreatic neuroendocrine tumors. Mechanistically, the CUL4B-DDB1-DCAF7 axis, activated by neddylation, enhances the ubiquitination and subsequent degradation of MEN1 at the K135, K151, K201, and K404 residues, therefore contributing to everolimus resistance [82]. Contrariwise, neddylation inactivation ameliorates the resistance of vemurafenib in melanoma, highlighting its inhibitory role in BRAF inhibitors [83]. Additionally, UBE2M-mediated neddylation promotes the decay of multiple substrates of CRLs, thereby shrinking the sensitivity of PARP inhibitor niraparib in castration-resistant prostate cancer [84].

### Neddylation-mediated endocrine therapy resistance

Endocrine therapy prevents the growth of tumor cells by altering the hormone levels. Strikingly, neddylation is a trigger of endocrine therapy resistance in breast cancer. For instance, cullin neddylation-induced SOX2 overexpression results in tamoxifen resistance [43]. Furthermore, CAND1, a regulator of cullin-RING E3 ubiquitin ligases, prevents estrogen receptor α (ERα) from CUL2-mediated proteasomal degradation. This preservation of ERα stability in breast cancer with AKTIP loss contributes to the development of tamoxifen resistance [85]. Additionally, overactivated neddylation facilitates the decay of serum and glucocorticoid-induced protein kinase (SGK), which further impedes nuclear export of FOXO3a, transcriptionally upregulating *ESR1* expression and leading to fulvestrant resistance [86].

### Neddylation-mediated immunotherapy resistance

Tumor immunotherapy activates or enhances the immune surveillance, thereby enabling the recognition and destruction of cancer cells. However, neddylation propels immune evasion *via* dysregulating the cytotoxicity of T cells [52], impeding the activity of NK cells [55], and intensifying the infiltration of tumor immunosuppressive cells [56], ultimately contributing to immunotherapy resistance. Moreover, mutations that destabilize the DNA mismatch repair (dMMR) system lead to proteome instability in dMMR tumors, accumulating misfolded proteins. In response, dMMR cells activate a NEDD8-dependent degradation pathway to clear these misfolded proteins. Therefore, inhibition of the NEDD8-mediated clearance pathway leads to the buildup of misfolded protein aggregates, which triggers immunogenic cell death and enhances the efficacy of PD1 inhibition in dMMR cancers [87]. In glioblastoma, neddylation reduces the efficacy of immune checkpoint blockade by regulating PD-L1 levels [57]. However, neddylation enhances immunotherapy sensitivity by promoting SHP2 neddylation in CRC [58].

Neddylation serves as a “double-edged sword” in tumor therapeutic resistance, likely due to tumor heterogeneity, treatment specificity, and microenvironmental stress. Consequently, neddylation-targeted therapies should incorporate individualized strategies and combination regimens to achieve optimal therapeutic efficacy.

### Antitumor potential of neddylation inhibitors

Malfunctions of neddylation catalytic enzymes frequently occur in tumors [22, 26, 65, 88], exerting a crucial effect on the cancer hallmark sustenance and anti-tumor drug sensitivity. This indicates that neddylation blockage holds significant potential for cancer treatment. Consequently, diverse neddylation inhibitors have been reported, including NAE inhibitors, E2-UBE2M-DCN1 inhibitors, E2-UBE2F inhibitors, and neddylation E3 inhibitors. However, except for MLN4924 (pevonedistat), all other inhibitors remain in the preclinical phase (Table 4).

**Table 4:** Inhibitors of neddylation molecules.

Compound	Target	R&D stage	Cancer type	Compound type	Refs
MLN4924	E1	Phase I/II/III	Solid and hematological malignancies	Adenosine sulfamate analog	[89]
ABP 1	E1	Preclinical	Lung, cervical, prostatic, and breast cancers	Adenosine sulfamate analog	[175]
ABP A3	E1	Preclinical	Solid and hematological malignancies	Adenosine sulfamate analog	[176]
TAS4464	E1	Phase I	AML, lymphoma, multiple myeloma, CLL, CRC, SCLC, sarcoma	Adenosine sulfamate analog	[108]
ZM223	E1	Preclinical	Colon cancer, osteosarcoma	Benzothiazole derivative	[177]
HA-1141	E1	Preclinical	Lung cancer	Non-adenosine sulfamate analog	[178]
6,6”-biapigenin	E1	Preclinical	CRC	Natural compound	[110]
Flavokawain A	E1	Preclinical	Prostate, breast, renal, liver, lung, colon and cervical cancers, melanoma, osteosarcoma	Natural compound	[111]
Flavokawain B	E1	Preclinical	Prostate cancer	Natural compound	[112]
Gartanin	E1	Preclinical	Prostate cancer	Natural compound	[113]
Piperacillin 1	E1	Preclinical	CRC	β-Lactam antibiotics	[114]
Micafungin	E2	Preclinical	GC	Antifungal agent	[121]
Arctigenin	E2	Preclinical	Lung cancer, GC	Natural compound	[122]
WS-299	E2	Preclinical	GC	Pyrimidine-based compound	[123]
HA-9104	E2	Preclinical	Lung cancer	UBE2F inhibitor	[74]
DI-591	UBE2M-DCN1	Preclinical	Esophageal cancer	Peptidomimetic inhibitor	[127]
DI-404	UBE2M-DCN1	Preclinical	Lung cancer	Peptidomimetic inhibitor	[128]
DI-1859	UBE2M-DCN1	Preclinical	Breast, esophageal, colon cancers	Peptidomimetic inhibitor	[129]
NAcM-HIT	UBE2M-DCN1	Preclinical	Lung and tongue squamous cell carcinoma	Piperidinyl urea-based inhibitor	[130]
Compound 49	UBE2M-DCN1	Preclinical	Lung cancer	Piperidinyl urea-based inhibitor	[131]
Compound 52	UBE2M-DCN1	Preclinical	Lung cancer	Piperidinyl urea-based inhibitor	[131]
NAcM-OPT	UBE2M-DCN1	Preclinical	Lung squamous cell carcinoma,	Piperidinyl urea-based inhibitor	[132]
Compound 1	UBE2M-DCN1	Preclinical	Lung and tongue squamous cell carcinoma	Pyrazolo-pyridone compound	[133]
Compound 27	UBE2M-DCN1	Preclinical	Lung and tongue squamous cell carcinoma	Pyrazolo-pyridone compound	[133]
Compound 40	UBE2M-DCN1	Preclinical	Lung squamous cell carcinoma	Pyrazolo-pyridone compound	[134]
WS-291	UBE2M-DCN1	Preclinical	GC	Pyrimidine-based compound	[135]
WS-383	UBE2M-DCN1	Preclinical	GC	Pyrimidine-based compound	[135]
DC-1	UBE2M-DCN1	Preclinical	Esophageal, liver, prostatic, breast cancers	Pyrimidine-based compound	[136]
DC-2	UBE2M-DCN1	Preclinical	Esophageal, liver, prostatic, breast cancers	Pyrimidine-based compound	[136]
DN-2	UBE2M-DCN1	Preclinical	Unkown	Pyrimidine-based compound	[137]
C64	E3	Preclinical	Pan-cancer	Polyheterocyclic derivative	[138]
Gossypol	E3	Preclinical	Lung, cervical, prostatic, breast cancers, leukemia, myeloma	Natural compound	[139]

### Neddylation NAE inhibitors

MLN4924, the first-in-class small-molecule inhibitor of NAE, selectively impedes the NAE enzyme activity by establishing a covalent NEDD8-MLN4924 adduct at the NAE active site, therefore inactivating all CRLs and accumulating CRL substrates [89, 90]. Numerous preclinical studies have illustrated that MLN4924 dampens tumor malignant traits [91**–**94] and sensitizes tumor cells to anti-tumor therapies [95**–**98]. Multiple clinical trials registered on ClinicalTrials.gov have been initiated to evaluate the therapeutic efficacy and safety of MLN4924 in cancer patients (Table 5).

**Table 5:** Clinical trials investigating MLN4924 as monotherapy and polytherapy in cancer treatment.

Mono-/poly-therapy	NCT number	Phase	Interventional model	Cancer	First posted	Study status
MLN4924	NCT00677170	I	Single Group Assignment	Solid tumors	2008	Completed
MLN4924	NCT00722488	I	Single Group Assignment	Lymphoma, MM	2008	Completed
MLN4924	NCT01011530	I	Single Group Assignment	Metastatic melanoma	2009	Completed
Azacitidine	NCT00911066	I	Single Group Assignment	AML, MDS, ALL	2009	Completed
Etoposide, Prednisone, Cyclophosphamide, Rituximab, Filgrastim Doxorubicin, Vincristine	NCT01415765	I/II	Single Group Assignment	Large B-cell lymphoma	2011	Withdrawn
Paclitaxel, Gemcitabine, Docetaxel, Carboplatin	NCT01862328	I	Sequential Assignment	Solid tumors	2013	Completed
Azacitidine	NCT01814826	I	Sequential Assignment	AML	2013	Completed
Fluconazole, Itraconazole, Docetaxel, Carboplatin, Paclitaxel	NCT02122770	I	Parallel Assignment	Advanced solid tumors	2014	Completed
Azacitidine	NCT02610777	II	Parallel Assignment	Higher-risk MDS, CML, or AML	2015	Completed
Azacitidine	NCT02782468	I	Parallel Assignment	AML, MDS	2016	Completed
Cytarabine, Idarubicin	NCT03330821	I/II	Single Group Assignment	AML	2017	Active, not recruiting
Irinotecan, Temozolomide	NCT03323034	I	Single Group Assignment	Relapsed or refractory solid tumors or lymphoma	2017	Completed
Pemetrexed, cisplatin	NCT03319537	I/II	Sequential Assignment	Mesothelioma	2017	Terminated
Docetaxel, Carboplatin, Paclitaxel	NCT03057366	I	Sequential Assignment	Advanced solid tumors	2017	Completed
Azacitidine	NCT03238248	II	Single Group Assignment	MDS or MPN	2017	Completed
Decitabine	NCT03009240	I	Single Group Assignment	AML	2017	Active, not recruiting
Docetaxel, Carboplatin, Paclitaxel	NCT03330106	I	Crossover Assignment	Advanced solid tumors	2017	Completed
Docetaxel	NCT03228186	II	Single Group Assignment	NSCLC	2017	Terminated
Vincristine, Dexamethasone, PEG-asparaginase, Doxorubicin, Methotrexate, Hydrocortisone, Cytarabine	NCT03349281	I	Parallel Assignment	Relapsed/Refractory ALL or L-NHL	2017	Completed
Azacitidine	NCT03268954	III	Parallel Assignment	Higher-risk MDS, CML, or AML	2017	Completed
Azacitidine	NCT03745352	II	Parallel Assignment	Relapsed or refractory AML	2018	Withdrawn
Ixazomib Citrate	NCT03770260	I	Single Group Assignment	MM	2018	Completed
Azacitidine, transplant	NCT03709576	II	Single Group Assignment	AML	2018	Terminated
Belinostat	NCT03772925	I	Single Group Assignment	Relapsed or refractory AML or MDS	2018	Terminated
Ibrutinib	NCT03479268	I	Single Group Assignment	Relapsed or refractory CLL and NHL	2018	Active, not recruiting
Cytarabine	NCT03459859	I	Parallel Assignment	AML, MDS	2018	Completed
Rifampin, Docetaxel, Carboplatin, Paclitaxel	NCT03486314	I	Sequential Assignment	Advanced solid tumors	2018	Completed
Carboplatin, Paclitaxel	NCT03965689	II	Single Group Assignment	NSCLC	2019	Active, not recruiting
Azacitidine, Cytarabine, Fludarabine Phosphate, Methotrexate, Therapeutic Hydrocortisone	NCT03813147	I	Single Group Assignment	Relapsed or refractory AML or MDS	2019	Completed
Carboplatin, Paclitaxel	NCT04175912	II	Parallel Assignment	Bile duct cancer	2019	Active, not recruiting
Azacitidine, Venetoclax	NCT03862157	I/II	Single Group Assignment	AML	2019	Active, not recruiting
Azacitidine, Venetoclax	NCT04172844	I	Sequential Assignment	AML	2019	Terminated
Azacitidine	NCT03814005	I	Sequential Assignment	Blood cancers or solid tumors with kidney or liver problems	2019	Completed
Azacitidine	NCT04090736	III	Parallel Assignment	AML	2019	Active, not recruiting
Venetoclax, Azacitidine	NCT04266795	II	Parallel Assignment	AML	2020	Active, not recruiting
Pembrolizumab	NCT04800627	I/II	Single Group Assignment	dMMR/MSI-H metastatic or locally advanced unresectable solid tumor	2021	Terminated
Azacitidine	NCT04712942	II	Parallel Assignment	AML, MDS	2021	Completed
Decitabine, Cedazuridine	NCT04985656	II	Single Group Assignment	MDS	2021	Withdrawn

*AML* Acute myelogenous leukemia, *MDS* Myelodysplastic syndrome, *ALL* Acute lymphoblastic leukemia, *CLL* Chronic lymphocytic leukemia, *NHL* Non-Hodgkin lymphoma, *MPN* Myelodysplastic/myeloproliferative overlap syndromes, *L-NHL* Lymphoblastic Non-Hodgkin Lymphoma, *CML* Chronic myelomonocytic leukemia, *MM* Multiple myeloma.

Uncontrolled tumor cell proliferation is a hallmark of cancer, with cell cycle dysregulation in tumorigenesis [32]. MLN4924 inhibits tumor growth by arresting the cell cycle. It accumulates CRL substrates, involving p21, p27, and CDT1, leading to G2 cell-cycle arrest and suppressing tumor cell proliferation in glioblastoma [99]. Likewise, MLN4924 triggers cell cycle arrest at the G2/M phase by reducing phospho-histone H3 and enhancing phospho-cdc2 levels, leading to the growth inhibition of urothelial carcinoma [100]. Cellular senescence, a permanent and irreversible cessation of the cell cycle and cell division, plays a significant role in the pathological processes of cancer [101]. MLN4924 impedes tumor growth by promoting p21-mediated cellular senescence. Specifically, MLN4924 inhibits the activities of CRL/SCF E3 ubiquitin ligases, involving skp2 and CRL4, thus accumulating p21 and promoting senescence in CRC, lung cancer, glioblastoma, and lymphoma [102**–**104]. MLN4924 typically eliminates tumor cells through PCD mechanisms. For example, MLN4924-induced CRL/SCFβTrcp stabilizes ATF4, thus stimulating the extrinsic apoptosis *via* the CHOP-DR5 axis in esophageal cancer cells [34]. In liver cancer cells, MLN4924 triggers autophagy and apoptosis by cullin neddylation blockage and CRL inactivation [38]. Furthermore, MLN4924 inhibits tumor angiogenesis and eliminates CSCs [26, 80]. Remarkably, MLN4924 also enhances the sensitivity of tumor cells to anti-cancer therapies. For instance, MLN4924 sensitizes CRC to topoisomerase I inhibitors by inactivating the DCAF13-CRL4 ubiquitin ligase complex [97]. Collectively, these findings indicate that MLN4924 exerts its antitumor effects by targeting the neddylation-CRL pathway, regulating the stability of tumor-related proteins, and holds potential for clinical applications. However, MLN4924 also exhibits potential tumor-promoting effects. It transcriptionally increases *PD-L1* gene expression via upregulating c-MYC, contributing to immune evasion in glioblastoma [57]. Hence, combining MLN4924 with specific target inhibitors could decrease the side effects and prevent the accumulation of cancer-promoting CRL substrates [105].

Besides MLN4924, multiple covalent and non-covalent NAE inhibitors have been identified and evaluated. TAS4464, a selective NAE inhibitor, forms a covalent adduct with NEDD8 within NAE through its sulfonamide structure in an ATP-dependent manner, restricting cullin neddylation and accumulating CRL substrates [106]. Preclinical in vitro and in vivo studies demonstrate TAS4464’s antitumor efficacy in hematologic and solid tumors [106**–**108]. However, an open-label, phase I clinical trial of TAS4464 in patients with advanced/metastatic solid tumors was discontinued due to its liver toxicity [109]. Multiple non-covalent NAE inhibitors, encompassing natural and synthetic compounds, have been identified and characterized. The second inhibitor of NAE 6,6”-biapigenin, a natural product-like inhibitor, creates hydrogen bonds with the C-terminal Glycine of NEDD8 and the side chain of Arg15 in APPBP1, disturbing the neddylation cascade [110]. Other natural compounds and FDA-approved drugs, such as Flavokawain A and B extracted from kava roots [111, 112], Gartanin identified from the purple mangosteen fruit [113], and FDA-approved drug piperacillin 1 [114], also interact with NAE and impede its enzymatic activity. However, all these inhibitors are merely in the preclinical phase, lacking clinical safety and efficacy evaluation.

### Neddylation E2 inhibitors

In mammals, UBE2M and UBE2F are the merely two known neddylation E2 conjugating enzymes, responsible for transferring NEDD8 from NAE to the E2 cysteine active site. Blocking the E2 enzyme activities prevents the binding of NEDD8 to target proteins. Therefore, neddylation E2 inhibitors hold promise as a therapeutic strategy in cancer treatment. UBE2M partners with RBX1 to facilitate the neddylation of CUL1, CUL2, CUL3, CUL4A, and CUL4B, activating the CRLs and manipulating their substrate levels [115]. UBE2M is aberrant in various tumors, and its blockade impedes malignancy [33, 116–120]. Micafungin, an antifungal agent, hinders the conjunction of NEDD8 to UBE2M and disrupts the neddylation cascade. Micafungin forms hydrogen bonds with UBE2M, particularly with its Glu107 and Asn109, suggesting that it may potentially disrupt the catalytic cysteine (Cys111) of UBE2M. In GC cells, Micafungin accumulates CRL substrates, therefore impeding malignant phenotypes and inducing DNA damage [121]. Arctigenin, a natural compound, effectively suppresses UBC12 enzyme activity, reducing cullin neddylation and abrogating tumor progression [122]. Additionally, WS-299 specifically hinders CUL3/5 neddylation by disrupting the PPI between RBX1 and UBE2M, leading to NOXA-mediated apoptosis in GC [123].

Analogous to UBE2M, UBE2F, possessing an N-terminal extension with a conserved catalytic core region, activates CRLs by catalyzing cullin neddylation. UBE2F contributes to tumor progression, making it a potential target for anticancer therapy. Overexpressed UBE2F enhances the growth, survival, and platinum-insensitivity of tumor cells. Mechanistically, UBE2F, integrating with RBX2, facilitates CUL5 neddylation, activating CRL5 and leading to NOXA depletion and apoptosis escape [35, 36, 64]. HA-9104, a small-molecule inhibitor targeting UBE2F, decreases UBE2F protein levels, thus boosting tumor cell apoptosis and radiosensitization through the accumulation of NOXA and deceleration of DNA damage repair [74].

Collectively, the development of high-throughput screening assays has advanced the identification of inhibitors targeting the “undruggable” E2 conjugating enzymes. Nevertheless, their clinical potential still needs to be thoroughly assessed.

### UBE2M-DCN1 inhibitors

Defective in cullin neddylation 1 (DCN1), an indispensable scaffold-like co-E3 ligase in the neddylation process, is dysregulated in multiple tumors [124, 125]. DCN1 directly interacts with UBE2M at a region that coincides with the E1-binding site, therefore facilitating the activation of CRLs by selectively catalyzing cullin neddylation and governing the activity of substrate proteins [126]. Consequently, disturbing UBE2M-DCN1 interaction presents another strategy for dampening the neddylation cascade. Three peptidomimetic derivatives, including DI-591, DI-404, and DI-1859, have been identified as selective inhibitors of the UBE2M-DCN1 interaction. DI-591, a high-affinity, cell-permeable small-molecule inhibitor that effectively blocks the interaction between DCN1 and UBE2M, selectively transforms cellular CUL3 into an un-neddylated, inactive form, with minimal or no effect on other cullin family members, contributing to a dose-dependent accumulation of NRF2 across various cancer cell lines [127]. DI-404, a further optimized small-molecule inhibitor derived from DI-591, targets the active sites of DCN1 more precisely. Similar to DI-591, DI-404 effectively and selectively blocks UBE2M-DCN1 interaction and CUL3 neddylation in several cancer cell lines [128]. DI-1548 and DI-1859, two potent covalent inhibitors targeting DCN1, specifically inhibit CUL3 neddylation without significantly affecting other cullins, and robustly increase NRF2 protein levels in the mouse liver. Importantly, DI-1859 effectively reduces acetaminophen-induced liver toxicity in mice [129].

Several piperidinyl urea derivatives have been identified as UBE2M-DCN1 inhibitors. NAcM-HIT specifically prevents the binding of DCN1 to UBE2M *via* occupying the N-acetyl-methionine binding pocket of DCN1, hindering DCN1-dependent cullin neddylation [130]. Compounds 49 and 52, analogs of NAcM-HIT, demonstrate a 100-fold increase in biochemical potency with improved solubility and permeability, though exhibiting low stability. These analogs selectively decline CUL1 and CUL3 neddylation levels, demonstrating the potential for anti-tumor activity [131]. Likewise, NAcM-OPT, an orally bioavailable inhibitor with satisfactory solubility and permeability, selectively decreases CUL1 and CUL3 neddylation levels and restrains cancer hallmarks in the squamous cell carcinoma line [132].

Through optimization of pyrazolo-pyridone compounds, a new category of inhibitors featuring a pyrazolo-pyridone core structure is developed, proficiently impeding UBE2M-DCN1 interaction. These pyrazolo-pyridone derivatives contain two chiral centers, which enhance their three-dimensional complexity, potentially leading to stronger binding affinity, greater target specificity, and enhanced solubility. Among them, compound 27 exhibits 25-fold greater potency than the initial hit, effectively stabilizing DCN1 and specifically decreasing the CUL1 and CUL3 neddylation levels in tumor cells [133]. Subsequently, compound 40 is identified to hinder UBE2M-DCN1 interaction with enhanced oral bioavailability, hampering tumor cell expansion [134].

Pyrimidine-based UBE2M-DCN1 inhibitors have also been discovered, including WS-291, WS-383, and triazolo[1,5-a]pyrimidine-based inhibitors. WS-383 specifically inhibits CUL1 and CUL3 neddylation *via* thwarting the UBE2M-DCN1 interaction, thus accumulating p21, p27, and NRF2 [135]. Two novel pyrimidin-based small molecular inhibitors, DC-1 and DC-2, have been unearthed. DC-2, a thiazole-containing 5-cyano-6-phenylpyrimidin-based inhibitor, selectively hinders UBE2M-DCN1 interaction, reducing CUL3 neddylation and accumulating NRF2 [136]. Compound DN-2, a 2-(Benzylthio)pyrimidine-based inhibitor targeting DCN1, is derived from structure-based optimizations [137]. While no UBE2M-DCN1 inhibitors have entered clinical trials, they offer valuable insights for future drug discovery.

### Neddylation E3 inhibitors

Neddylation E3 ligases determine the specificity of target substrates and assist in transferring NEDD8 from the E2 to the substrates. Therefore, targeting neddylation E3s holds potential for enhancing selectivity and reducing side effects. However, inhibitors targeting neddylation E3 ligases remain scarce. RBX1 and RBX2/SAG are E3 ligases involved in the neddylation. RBX1 works with UBE2M to facilitate the neddylation of CUL1-4, while RBX2/SAG collaborates with UBE2F to mediate the neddylation of CUL5 [7]. C64, a small-molecule inhibitor, preferentially binds to the “VLYRLWLN” structural motif at the RBX1-binding grooves of CUL scaffolds, impairing RBX1-CULs interaction and impeding cancer cell survival [138]. Moreover, gossypol, a natural compound derived from cottonseed, hinders the neddylation of CUL1 and CUL5 by directly binding to the RBX1-CUL1 or SAG-CUL5 complex, contributing to NOXA and MCL1 accumulation and tumor growth inhibition [139]. Advancing highly specific neddylation E3 inhibitors is crucial for effective pathway disruption.

## Conclusions and perspective

NEDD8 covalently modifies substrate lysine, regulating protein stability, function, and localization. Neddylation targets include cullins and non-cullin proteins. Cullin neddylation induces conformational alterations that remove CAND1, enabling CRL activation. This requires NEDD8 binding to a C-terminal cullin lysine, promoting functional CRL assembly and subsequent substrate ubiquitination [140, 141]. Neddylation of non-cullin substrates influences their stability, activity, or PPI. Nowadays, several bioinformatics tools that predict protein neddylation sites using machine learning have been developed, such as DTL-NeddSite (http://dtl-neddsite.bioinfogo.org/) and NeddPred (123.206.31.171/NeddPred/), helping researchers identify potential interaction sites on neddylated proteins. Neddylation mechanisms remain elusive. Some unresolved questions include: How does neddylation precisely select specific substrates? What other non-cullin proteins can undergo neddylation? Beyond the well-known E1, E2, and E3 enzymes, what other enzymes participate in the neddylation cascade? Most importantly, is targeting neddylation for anticancer therapy both safe and effective? Additionally, neddylation does not function in isolation but exhibits intricate crosstalk with other ubiquitin-like modifications (UBLs), particularly ubiquitination and SUMOylation. Recent advances in neddylation proteomics have uncovered the formation of hybrid NEDD8-ubiquitin and NEDD8-SUMO2 chains [142], indicating a previously unrecognized layer of post-translational regulation. These hybrid chains may serve as unique molecular signals, fine-tuning protein stability, localization, and functional interactions in ways distinct from canonical modifications, providing novel molecular mechanisms for proteostasis. Neddylation dysregulation continually occurs in malignancies, exhibiting dual roles in promoting and suppressing tumor progression. Remarkably, neddylation, a non-mutational epigenetic modification, is activated by diverse cellular stress and sustains cancer hallmarks while modulating therapy sensitivity. Therefore, targeting the neddylation enzymes is a potential strategy for anti-tumor treatment. Several small-molecule inhibitors have been established to target neddylation. Representatively, MLN4924 inhibits neddylation, suppressing tumor malignancy, and has entered phase I-III clinical trials. However, MLN4924 also exerts a tumor-promoting effect in some contexts. Since the neddylation cascade participates in essential physiological processes, its inhibition may cause on-target toxicity in normal cells. To address these challenges, the multidisciplinary development of selective neddylation inhibitors is crucial for effective cancer treatment.

## References

[1] Kamitani T, Kito K, Nguyen HP, Yeh ET. Characterization of NEDD8, a developmentally down-regulated ubiquitin-like protein. J Biol Chem. 1997;272:28557–62. 9353319 10.1074/jbc.272.45.28557

[2] Rabut G, Peter M. Function and regulation of protein neddylation. ‘Protein modifications: beyond usual suspects’ Rev Ser EMBO Rep. 2008;9:969–76. 10.1038/embor.2008.183 PMC2572130 18802447

[3] Enchev RI, Schulman BA, Peter M. Protein neddylation: beyond cullin-RING ligases. Nat Rev Mol Cell Biol. 2015;16:30–44. 25531226 10.1038/nrm3919 PMC5131867

[4] Zhou L, Jiang Y, Luo Q, Li L, Jia L. Neddylation: a novel modulator of the tumor microenvironment. Mol Cancer. 2019;18:77. 30943988 10.1186/s12943-019-0979-1 PMC6446326

[5] Yu M, Qian X, Wang Y, Li Q, Peng C, Chen B, et al. Emerging role of NEDD8-mediated neddylation in age-related metabolic diseases. Ageing Res Rev. 2024;94:102191. 38199526 10.1016/j.arr.2024.102191

[6] Sun J, Liu C, Lang C, Wang J, Li Q, Peng C, et al. Inhibiting neddylation: a new strategy for tumor therapy. J Pharm Anal. 2025;15:101140. 40496066 10.1016/j.jpha.2024.101140 PMC12151199

[7] Zhang S, Yu Q, Li Z, Zhao Y, Sun Y. Protein neddylation and its role in health and diseases. Signal Transduct Target Ther. 2024;9:85. 38575611 10.1038/s41392-024-01800-9 PMC10995212

[8] Kumar S, Tomooka Y, Noda M. Identification of a set of genes with developmentally down-regulated expression in the mouse brain. Biochem Biophys Res Commun. 1992;185:1155–61. 1378265 10.1016/0006-291x(92)91747-e

[9] Kumar S, Yoshida Y, Noda M. Cloning of a cDNA which encodes a novel ubiquitin-like protein. Biochem Biophys Res Commun. 1993;195:393–9. 8395831 10.1006/bbrc.1993.2056

[10] Bohnsack RN, Haas AL. Conservation in the mechanism of Nedd8 activation by the human AppBp1-Uba3 heterodimer. J Biol Chem. 2003;278:26823–30. 12740388 10.1074/jbc.M303177200

[11] Huang DT, Ayrault O, Hunt HW, Taherbhoy AM, Duda DM, Scott DC, et al. E2-RING expansion of the NEDD8 cascade confers specificity to cullin modification. Mol Cell. 2009;33:483–95. 19250909 10.1016/j.molcel.2009.01.011 PMC2725360

[12] Mendoza HM, Shen LN, Botting C, Lewis A, Chen J, Ink B, et al. NEDP1, a highly conserved cysteine protease that deNEDDylates Cullins. J Biol Chem. 2003;278:25637–43. 12730221 10.1074/jbc.M212948200

[13] Yang X, Menon S, Lykke-Andersen K, Tsuge T, Di X, Wang X, et al. The COP9 signalosome inhibits p27(kip1) degradation and impedes G1-S phase progression via deneddylation of SCF Cul1. Curr Biol. 2002;12:667–72. 11967155 10.1016/s0960-9822(02)00791-1

[14] Gong L, Kamitani T, Millas S, Yeh ET. Identification of a novel isopeptidase with dual specificity for ubiquitin- and NEDD8-conjugated proteins. J Biol Chem. 2000;275:14212–6. 10799498 10.1074/jbc.275.19.14212

[15] Wada H, Kito K, Caskey LS, Yeh ET, Kamitani T. Cleavage of the C-terminus of NEDD8 by UCH-L3. Biochem Biophys Res Commun. 1998;251:688–92. 9790970 10.1006/bbrc.1998.9532

[16] He ZX, Yang WG, Zengyangzong D, Gao G, Zhang Q, Liu HM, et al. Targeting cullin neddylation for cancer and fibrotic diseases. Theranostics. 2023;13:5017–56. 37771770 10.7150/thno.78876 PMC10526667

[17] Wu K, Yamoah K, Dolios G, Gan-Erdene T, Tan P, Chen A, et al. DEN1 is a dual function protease capable of processing the C terminus of Nedd8 and deconjugating hyper-neddylated CUL1. J Biol Chem. 2003;278:28882–91. 12759363 10.1074/jbc.M302888200

[18] Walden H, Podgorski MS, Schulman BA. Insights into the ubiquitin transfer cascade from the structure of the activating enzyme for NEDD8. Nature. 2003;422:330–4. 12646924 10.1038/nature01456

[19] Walden H, Podgorski MS, Huang DT, Miller DW, Howard RJ, Minor DL Jr., et al. The structure of the APPBP1-UBA3-NEDD8-ATP complex reveals the basis for selective ubiquitin-like protein activation by an E1. Mol Cell. 2003;12:1427–37. 14690597 10.1016/s1097-2765(03)00452-0

[20] Hori T, Osaka F, Chiba T, Miyamoto C, Okabayashi K, Shimbara N, et al. Covalent modification of all members of human cullin family proteins by NEDD8. Oncogene. 1999;18:6829–34. 10597293 10.1038/sj.onc.1203093

[21] Petillo S, Sproviero E, Loconte L, Cuollo L, Zingoni A, Molfetta R, et al. NEDD8-activating enzyme inhibition potentiates the anti-myeloma activity of natural killer cells. Cell Death Dis. 2023;14:438. 37460534 10.1038/s41419-023-05949-z PMC10352239

[22] Zhang F, Xiong X, Li Z, Wang H, Wang W, Zhao Y, et al. RHEB neddylation by the UBE2F-SAG axis enhances mTORC1 activity and aggravates liver tumorigenesis. EMBO J. 2025;44:1185–219. 39762645 10.1038/s44318-024-00353-5 PMC11832924

[23] Guo YJ, Pang JR, Zhang Y, Li ZR, Zi XL, Liu HM, et al. Neddylation-dependent LSD1 destabilization inhibits the stemness and chemoresistance of gastric cancer. Int J Biol Macromol. 2024;254:126801. 37689288 10.1016/j.ijbiomac.2023.126801

[24] Jiang Y, Liang Y, Li L, Zhou L, Cheng W, Yang X, et al. Targeting neddylation inhibits intravascular survival and extravasation of cancer cells to prevent lung-cancer metastasis. Cell Biol Toxicol. 2019;35:233–45. 31140025 10.1007/s10565-019-09472-w

[25] Wolf ER, Mabry AR, Damania B, Mayo LD. Mdm2-mediated neddylation of pVHL blocks the induction of antiangiogenic factors. Oncogene. 2020;39:5228–39. 32555333 10.1038/s41388-020-1359-4 PMC7368819

[26] Jin Y, Zhang P, Wang Y, Jin B, Zhou J, Zhang J, et al. Neddylation blockade diminishes hepatic metastasis by dampening cancer stem-like cells and angiogenesis in uveal melanoma. Clin Cancer Res. 2018;24:3741–54. 29233905 10.1158/1078-0432.CCR-17-1703

[27] Yao WT, Wu JF, Yu GY, Wang R, Wang K, Li LH, et al. Suppression of tumor angiogenesis by targeting the protein neddylation pathway. Cell Death Dis. 2014;5:e1059. 24525735 10.1038/cddis.2014.21 PMC3944239

[28] Zhu T, Wang J, Pei Y, Wang Q, Wu Y, Qiu G, et al. Neddylation controls basal MKK7 kinase activity in breast cancer cells. Oncogene. 2016;35:2624–33. 26364603 10.1038/onc.2015.323

[29] Jiang Y, Le F, Huang S, Chen X, Deng Z. MLN4924 Suppresses head and neck squamous cell carcinoma progression by inactivating the mTOR signaling pathway via the NEDD8/CUL4/TSC2 axis. Int J Biochem Cell Biol. 2024;177:106696. 39566655 10.1016/j.biocel.2024.106696

[30] Park JB, Seo J, Park JW, Chun YS. Neddylation blockade induces HIF-1alpha driven cancer cell migration via upregulation of ZEB1. Sci Rep. 2020;10:18210. 33097763 10.1038/s41598-020-75286-0 PMC7585416

[31] Kim Y, Park JB, Fukuda J, Watanabe M, Chun YS. The effect of neddylation blockade on slug-dependent cancer cell migration is regulated by p53 mutation status. Cancers. 2021;13:531. 33573293 10.3390/cancers13030531 PMC7866814

[32] Hanahan D, Weinberg RA. Hallmarks of cancer: the next generation. Cell. 2011;144:646–74. 21376230 10.1016/j.cell.2011.02.013

[33] Wang S, Xian J, Li L, Jiang Y, Liu Y, Cai L, et al. NEDD8-conjugating enzyme UBC12 as a novel therapeutic target in esophageal squamous cell carcinoma. Signal Transduct Target Ther. 2020;5:123. 32651357 10.1038/s41392-020-00226-3 PMC7351728

[34] Chen P, Hu T, Liang Y, Li P, Chen X, Zhang J, et al. Neddylation inhibition activates the extrinsic apoptosis pathway through ATF4-CHOP-DR5 axis in human esophageal cancer cells. Clin Cancer Res. 2016;22:4145–57. 26983464 10.1158/1078-0432.CCR-15-2254

[35] Zhou W, Xu J, Li H, Xu M, Chen ZJ, Wei W, et al. Neddylation E2 UBE2F promotes the survival of lung cancer cells by activating CRL5 to degrade NOXA via the K11 Linkage. Clin Cancer Res. 2017;23:1104–16. 27591266 10.1158/1078-0432.CCR-16-1585 PMC5315595

[36] Xu S, Ma Y, Tong Q, Yang J, Liu J, Wang Y, et al. Cullin-5 neddylation-mediated NOXA degradation is enhanced by PRDX1 oligomers in colorectal cancer. Cell Death Dis. 2021;12:265. 33712558 10.1038/s41419-021-03557-3 PMC7954848

[37] Luo Z, Pan Y, Jeong LS, Liu J, Jia L. Inactivation of the Cullin (CUL)-RING E3 ligase by the NEDD8-activating enzyme inhibitor MLN4924 triggers protective autophagy in cancer cells. Autophagy. 2012;8:1677–9. 22874562 10.4161/auto.21484 PMC3494597

[38] Luo Z, Yu G, Lee HW, Li L, Wang L, Yang D, et al. The Nedd8-activating enzyme inhibitor MLN4924 induces autophagy and apoptosis to suppress liver cancer cell growth. Cancer Res. 2012;72:3360–71. 22562464 10.1158/0008-5472.CAN-12-0388

[39] Zhao Y, Xiong X, Jia L, Sun Y. Targeting cullin-RING ligases by MLN4924 induces autophagy via modulating the HIF1-REDD1-TSC1-mTORC1-DEPTOR axis. Cell Death Dis. 2012;3:e386. 22951983 10.1038/cddis.2012.125 PMC3461362

[40] Lv Y, Li B, Han K, Xiao Y, Yu X, Ma Y, et al. The Nedd8-activating enzyme inhibitor MLN4924 suppresses colon cancer cell growth via triggering autophagy. Korean J Physiol Pharm. 2018;22:617–25. 10.4196/kjpp.2018.22.6.617 PMC6205944 30402022

[41] Liang Y, Jiang Y, Jin X, Chen P, Heng Y, Cai L, et al. Neddylation inhibition activates the protective autophagy through NF-kappaB-catalase-ATF3 Axis in human esophageal cancer cells. Cell Commun Signal. 2020;18:72. 32398095 10.1186/s12964-020-00576-z PMC7218644

[42] Zhou Q, Yu H, Chen Y, Ren J, Lu Y, Sun Y. The CRL3(KCTD10) ubiquitin ligase-USP18 axis coordinately regulates cystine uptake and ferroptosis by modulating SLC7A11. Proc Natl Acad Sci USA. 2024;121:e2320655121. 38959043 10.1073/pnas.2320655121 PMC11252818

[43] Yin Y, Xie CM, Li H, Tan M, Chen G, Schiff R, et al. The FBXW2-MSX2-SOX2 axis regulates stem cell property and drug resistance of cancer cells. Proc Natl Acad Sci USA. 2019;116:20528–38. 31548378 10.1073/pnas.1905973116 PMC6789910

[44] Zhou X, Tan M, Nyati MK, Zhao Y, Wang G, Sun Y. Blockage of neddylation modification stimulates tumor sphere formation in vitro and stem cell differentiation and wound healing in vivo. Proc Natl Acad Sci USA. 2016;113:E2935–44. 27162365 10.1073/pnas.1522367113 PMC4889367

[45] Shamay M, Greenway M, Liao G, Ambinder RF, Hayward SD. De novo DNA methyltransferase DNMT3b interacts with NEDD8-modified proteins. J Biol Chem. 2010;285:36377–86. 20847044 10.1074/jbc.M110.155721 PMC2978566

[46] Chang Y, Chen Q, Li H, Xu J, Tan M, Xiong X, et al. The UBE2F-CRL5(ASB11)-DIRAS2 axis is an oncogene and tumor suppressor cascade in pancreatic cancer cells. Dev Cell. 2024;59:1317–32. 38574733 10.1016/j.devcel.2024.03.018

[47] Su Y, Luo Y, Zhang P, Lin H, Pu W, Zhang H, et al. Glucose-induced CRL4(COP1)-p53 axis amplifies glycometabolism to drive tumorigenesis. Mol Cell. 2023;83:2316–31. 37390815 10.1016/j.molcel.2023.06.010

[48] Zhou Q, Lin W, Wang C, Sun F, Ju S, Chen Q, et al. Neddylation inhibition induces glutamine uptake and metabolism by targeting CRL3(SPOP) E3 ligase in cancer cells. Nat Commun. 2022;13:3034. 35641493 10.1038/s41467-022-30559-2 PMC9156729

[49] Xie P, Peng Z, Chen Y, Li H, Du M, Tan Y, et al. Neddylation of PTEN regulates its nuclear import and promotes tumor development. Cell Res. 2021;31:291–311. 33299139 10.1038/s41422-020-00443-z PMC8027835

[50] Wu L, Jin Y, Zhao X, Tang K, Zhao Y, Tong L, et al. Tumor aerobic glycolysis confers immune evasion through modulating sensitivity to T cell-mediated bystander killing via TNF-alpha. Cell Metab. 2023;35:1580–96. 37506695 10.1016/j.cmet.2023.07.001

[51] Ganapathy-Kanniappan S. Linking tumor glycolysis and immune evasion in cancer: emerging concepts and therapeutic opportunities. Biochim Biophys Acta Rev Cancer. 2017;1868:212–20. 28400131 10.1016/j.bbcan.2017.04.002

[52] Liao X, Li W, Zhou H, Rajendran BK, Li A, Ren J, et al. The CUL5 E3 ligase complex negatively regulates central signaling pathways in CD8(+) T cells. Nat Commun. 2024;15:603. 38242867 10.1038/s41467-024-44885-0 PMC10798966

[53] Wang X, Chen C, Vuong D, Rodriguez-Rodriguez S, Lam V, Roleder C, et al. Pharmacologic targeting of Nedd8-activating enzyme reinvigorates T-cell responses in lymphoid neoplasia. Leukemia. 2023;37:1324–35. 37031300 10.1038/s41375-023-01889-x PMC10244170

[54] Best S, Lam V, Liu T, Bruss N, Kittai A, Danilova OV, et al. Immunomodulatory effects of pevonedistat, a NEDD8-activating enzyme inhibitor, in chronic lymphocytic leukemia-derived T cells. Leukemia. 2021;35:156–68. 32203139 10.1038/s41375-020-0794-0 PMC8288064

[55] Petillo S, Capuano C, Molfetta R, Fionda C, Mekhloufi A, Pighi C, et al. Immunomodulatory effect of NEDD8-activating enzyme inhibition in multiple myeloma: upregulation of NKG2D ligands and sensitization to natural killer cell recognition. Cell Death Dis. 2021;12:836. 34482362 10.1038/s41419-021-04104-w PMC8418610

[56] Lin X, Yang S, Zhou C, Ao C, Sun D. The NEDD8-activating enzyme E1 UBA3 orchestrates the immunosuppressive microenvironment in lung adenocarcinoma via the NF-small ka, CyrillicB pathway. Med Oncol. 2023;40:286. 37656220 10.1007/s12032-023-02162-y PMC10474176

[57] Zhou S, Zhao X, Yang Z, Yang R, Chen C, Zhao K, et al. Neddylation inhibition upregulates PD-L1 expression and enhances the efficacy of immune checkpoint blockade in glioblastoma. Int J Cancer. 2019;145:763–74. 31044422 10.1002/ijc.32379

[58] Li Y, Zhou H, Liu P, Lv D, Shi Y, Tang B, et al. SHP2 deneddylation mediates tumor immunosuppression in colon cancer via the CD47/SIRPalpha axis. J Clin Invest. 2023;133:e162870. 36626230 10.1172/JCI162870 PMC9927946

[59] Hughes DJ, Wood JJ, Jackson BR, Baquero-Perez B, Whitehouse A. NEDDylation is essential for Kaposi’s sarcoma-associated herpesvirus latency and lytic reactivation and represents a novel anti-KSHV target. PLoS Pathog. 2015;11:e1004771. 25794275 10.1371/journal.ppat.1004771 PMC4368050

[60] Liu N, Zhang J, Yang X, Jiao T, Zhao X, Li W, et al. HDM2 promotes NEDDylation of hepatitis B virus HBx to enhance its stability and function. J Virol. 2017;91:e00340-17. 28592528 10.1128/JVI.00340-17 PMC5533936

[61] Chen Y, Xian M, Ying W, Liu J, Bing S, Wang X, et al. Succinate dehydrogenase deficiency-driven succinate accumulation induces drug resistance in acute myeloid leukemia via ubiquitin-cullin regulation. Nat Commun. 2024;15:9820. 39537588 10.1038/s41467-024-53398-9 PMC11560925

[62] Yuan T, Zhou T, Qian M, Du J, Liu Y, Wang J, et al. SDHA/B reduction promotes hepatocellular carcinoma by facilitating the deNEDDylation of cullin1 and stabilizing YAP/TAZ. Hepatology. 2023;78:103–19. 35713976 10.1002/hep.32621

[63] Ma Y, Huang X, Wang Y, Lei Y, Yu J, Yu S, et al. NNMT/1-MNA promote cell-cycle progression of breast cancer by targeting UBC12/Cullin-1-mediated degradation of P27 proteins. Adv Sci. 2024;11:e2305907. 10.1002/advs.202305907 PMC10916551 38126621

[64] Zhou L, Zhu J, Chen W, Jiang Y, Hu T, Wang Y, et al. Induction of NEDD8-conjugating enzyme E2 UBE2F by platinum protects lung cancer cells from apoptosis and confers to platinum-insensitivity. Cell Death Dis. 2020;11:975. 33184273 10.1038/s41419-020-03184-4 PMC7665193

[65] Zheng B, Qian F, Wang X, Wang Y, Zhou B, Fang L. Neddylation activated TRIM25 desensitizes triple-negative breast cancer to paclitaxel via TFEB-mediated autophagy. J Exp Clin Cancer Res. 2024;43:177. 38926803 10.1186/s13046-024-03085-w PMC11201311

[66] Nawrocki ST, Kelly KR, Smith PG, Espitia CM, Possemato A, Beausoleil SA, et al. Disrupting protein NEDDylation with MLN4924 is a novel strategy to target cisplatin resistance in ovarian cancer. Clin Cancer Res. 2013;19:3577–90. 23633453 10.1158/1078-0432.CCR-12-3212

[67] Jazaeri AA, Shibata E, Park J, Bryant JL, Conaway MR, Modesitt SC, et al. Overcoming platinum resistance in preclinical models of ovarian cancer using the neddylation inhibitor MLN4924. Mol Cancer Ther. 2013;12:1958–67. 23939375 10.1158/1535-7163.MCT-12-1028 PMC3795967

[68] Funke K, Einsfelder U, Hansen A, Arevalo L, Schneider S, Nettersheim D, et al. Genome-scale CRISPR screen reveals neddylation to contribute to cisplatin resistance of testicular germ cell tumours. Br J Cancer. 2023;128:2270–82. 37024667 10.1038/s41416-023-02247-5 PMC10241889

[69] Lai QY, He YZ, Peng XW, Zhou X, Liang D, Wang L. Histone deacetylase 1 induced by neddylation inhibition contributes to drug resistance in acute myelogenous leukemia. Cell Commun Signal. 2019;17:86. 31358016 10.1186/s12964-019-0393-8 PMC6664585

[70] Yuan TZ, Lin HY, Kuei CH, Lin CH, Lee HH, Lee HL, et al. NEDD8 promotes radioresistance via triggering autophagy formation and serves as a novel prognostic marker in oral squamous cell carcinoma. Cancer Cell Int. 2023;23:41. 36890567 10.1186/s12935-023-02883-0 PMC9993556

[71] Wei D, Li H, Yu J, Sebolt JT, Zhao L, Lawrence TS, et al. Radiosensitization of human pancreatic cancer cells by MLN4924, an investigational NEDD8-activating enzyme inhibitor. Cancer Res. 2012;72:282–93. 22072567 10.1158/0008-5472.CAN-11-2866 PMC3251739

[72] Vanderdys V, Allak A, Guessous F, Benamar M, Read PW, Jameson MJ, et al. The neddylation inhibitor pevonedistat (MLN4924) suppresses and radiosensitizes head and neck squamous carcinoma cells and tumors. Mol Cancer Ther. 2018;17:368–80. 28838998 10.1158/1535-7163.MCT-17-0083 PMC5805645

[73] Shao Y, Liu Z, Song X, Sun R, Zhou Y, Zhang D, et al. ALKBH5/YTHDF2-mediated m6A modification of circAFF2 enhances radiosensitivity of colorectal cancer by inhibiting Cullin neddylation. Clin Transl Med. 2023;13:e1318. 37381158 10.1002/ctm2.1318 PMC10307995

[74] Xu T, Ma Q, Li Y, Yu Q, Pan P, Zheng Y, et al. A small molecule inhibitor of the UBE2F-CRL5 axis induces apoptosis and radiosensitization in lung cancer. Signal Transduct Target Ther. 2022;7:354. 36253371 10.1038/s41392-022-01182-w PMC9576757

[75] Zhou L, Dong C, Xu Z, Wang X, Zhang L, Chen S, et al. NEDD8-conjugating enzyme E2 UBE2F confers radiation resistance by protecting lung cancer cells from apoptosis. J Zhejiang Univ Sci B. 2021;22:959–65. 34783226 10.1631/jzus.B2100170 PMC8593528

[76] Deng T, Zhu Q, Xie L, Liu Y, Peng Y, Yin L, et al. Norcantharidin promotes cancer radiosensitization through Cullin1 neddylation-mediated CDC6 protein degradation. Mol Carcinog. 2022;61:812–24. 35652616 10.1002/mc.23435

[77] Zheng S, Wu Y, Li Z. Integrating cullin2-RING E3 ligase as a potential biomarker for glioblastoma multiforme prognosis and radiosensitivity profiling. Radiother Oncol. 2021;154:36–44. 32918970 10.1016/j.radonc.2020.09.005

[78] Guihard S, Ramolu L, Macabre C, Wasylyk B, Noel G, Abecassis J, et al. The NEDD8 conjugation pathway regulates p53 transcriptional activity and head and neck cancer cell sensitivity to ionizing radiation. Int J Oncol. 2012;41:1531–40. 22895816 10.3892/ijo.2012.1584

[79] Zhao M, Dai B, Li X, Zhang Y, Qiao C, Qin Y, et al. RAPSYN-mediated neddylation of BCR-ABL alternatively determines the fate of Philadelphia chromosome-positive leukemia. Elife. 2024;12. 10.7554/eLife.88375 PMC11168747 38865175

[80] Liu C, Nie D, Li J, Du X, Lu Y, Li Y, et al. Antitumor effects of blocking protein neddylation in T315I-BCR-ABL leukemia cells and leukemia stem cells. Cancer Res. 2018;78:1522–36. 29321163 10.1158/0008-5472.CAN-17-1733

[81] Bahjat M, de Wilde G, van Dam T, Maas C, Bloedjes T, Bende RJ, et al. The NEDD8-activating enzyme inhibitor MLN4924 induces DNA damage in Ph+ leukemia and sensitizes for ABL kinase inhibitors. Cell Cycle. 2019;18:2307–22. 31349760 10.1080/15384101.2019.1646068 PMC6738521

[82] Xu J, Ye Z, Zhuo Q, Gao H, Qin Y, Lou X, et al. MEN1 Degradation Induced by Neddylation and the CUL4B-DCAF7 Axis Promotes Pancreatic Neuroendocrine Tumor Progression. Cancer Res. 2023;83:2226–47. 36939378 10.1158/0008-5472.CAN-22-3599

[83] Huang X, Yi P, Gou W, Zhang R, Wu C, Liu L, et al. Neddylation signaling inactivation by tetracaine hydrochloride suppresses cell proliferation and alleviates vemurafenib-resistance of melanoma. Cell Biol Toxicol. 2024;40:81. 39297891 10.1007/s10565-024-09916-y PMC11413085

[84] Zhao X, Jiang L, Hu D, Tang Y, Zhao G, Du X, et al. NPRL2 reduces the niraparib sensitivity of castration-resistant prostate cancer via interacting with UBE2M and enhancing neddylation. Exp Cell Res. 2021;403:112614. 33905671 10.1016/j.yexcr.2021.112614

[85] Ng ASN, Zhang S, Mak VCY, Zhou Y, Yuen Y, Sharma R, et al. AKTIP loss is enriched in ERalpha-positive breast cancer for tumorigenesis and confers endocrine resistance. Cell Rep. 2022;41:111821. 36516775 10.1016/j.celrep.2022.111821 PMC9837615

[86] Jia X, Li C, Li L, Liu X, Zhou L, Zhang W, et al. Neddylation inactivation facilitates FOXO3a nuclear export to suppress estrogen receptor transcription and improve fulvestrant sensitivity. Clin Cancer Res. 2019;25:3658–72. 30833270 10.1158/1078-0432.CCR-18-2434

[87] McGrail DJ, Garnett J, Yin J, Dai H, Shih DJH, Lam TNA, et al. Proteome instability is a therapeutic vulnerability in mismatch repair-deficient cancer. Cancer Cell. 2020;37:371–86. 32109374 10.1016/j.ccell.2020.01.011 PMC7337255

[88] Zhang H, Yang J, Song Q, Ding X, Sun F, Yang L. UBA3 promotes the occurrence and metastasis of intrahepatic cholangiocarcinoma through MAPK signaling pathway. Acta Biochim Biophys Sin. 2024;56:199–209. 38298057 10.3724/abbs.2024014 PMC10984854

[89] Soucy TA, Smith PG, Milhollen MA, Berger AJ, Gavin JM, Adhikari S, et al. An inhibitor of NEDD8-activating enzyme as a new approach to treat cancer. Nature. 2009;458:732–6. 19360080 10.1038/nature07884

[90] Brownell JE, Sintchak MD, Gavin JM, Liao H, Bruzzese FJ, Bump NJ, et al. Substrate-assisted inhibition of ubiquitin-like protein-activating enzymes: the NEDD8 E1 inhibitor MLN4924 forms a NEDD8-AMP mimetic in situ. Mol Cell. 2010;37:102–11. 20129059 10.1016/j.molcel.2009.12.024

[91] Calandrini C, van Hooff SR, Paassen I, Ayyildiz D, Derakhshan S, Dolman MEM, et al. Organoid-based drug screening reveals neddylation as therapeutic target for malignant rhabdoid tumors. Cell Rep. 2021;36:109568. 34433038 10.1016/j.celrep.2021.109568

[92] Norton JP, Augert A, Eastwood E, Basom R, Rudin CM, MacPherson D. Protein neddylation as a therapeutic target in pulmonary and extrapulmonary small cell carcinomas. Genes Dev. 2021;35:870–87. 34016692 10.1101/gad.348316.121 PMC8168556

[93] Mittler F, Obeid P, Haguet V, Allier C, Gerbaud S, Rulina AV, et al. Mechanical stress shapes the cancer cell response to neddylation inhibition. J Exp Clin Cancer Res. 2022;41:115. 35354476 10.1186/s13046-022-02328-y PMC8966269

[94] Tan M, Li H, Sun Y. Endothelial deletion of Sag/Rbx2/Roc2 E3 ubiquitin ligase causes embryonic lethality and blocks tumor angiogenesis. Oncogene. 2014;33:5211–20. 24213570 10.1038/onc.2013.473 PMC4016996

[95] Zou L, Cao D, Sun Q, Yu W, Li B, Xu G, et al. The histone demethylase KDM5C enhances the sensitivity of acute myeloid leukemia cells to lenalidomide by stabilizing cereblon. Cell Mol Biol Lett. 2025;30:14. 39881283 10.1186/s11658-025-00697-8 PMC11780777

[96] Wong B, Bergeron A, Maznyi G, Ng K, Jirovec A, Birdi HK, et al. Pevonedistat, a first-in-class NEDD8-activating enzyme inhibitor, sensitizes cancer cells to VSVDelta51 oncolytic virotherapy. Mol Ther. 2023;31:3176–92. 37766429 10.1016/j.ymthe.2023.09.017 PMC10638453

[97] Sun Y, Baechler SA, Zhang X, Kumar S, Factor VM, Arakawa Y, et al. Targeting neddylation sensitizes colorectal cancer to topoisomerase I inhibitors by inactivating the DCAF13-CRL4 ubiquitin ligase complex. Nat Commun. 2023;14:3762. 37353483 10.1038/s41467-023-39374-9 PMC10290057

[98] Cojocari D, Smith BN, Purkal JJ, Arrate MP, Huska JD, Xiao Y, et al. Pevonedistat and azacitidine upregulate NOXA (PMAIP1) to increase sensitivity to venetoclax in preclinical models of acute myeloid leukemia. Haematologica. 2022;107:825–35. 33853293 10.3324/haematol.2020.272609 PMC8968901

[99] Hua W, Li C, Yang Z, Li L, Jiang Y, Yu G, et al. Suppression of glioblastoma by targeting the overactivated protein neddylation pathway. Neuro Oncol. 2015;17:1333–43. 25904638 10.1093/neuonc/nov066 PMC4578582

[100] Kuo KL, Ho IL, Shi CS, Wu JT, Lin WC, Tsai YC, et al. MLN4924, a novel protein neddylation inhibitor, suppresses proliferation and migration of human urothelial carcinoma: In vitro and in vivo studies. Cancer Lett. 2015;363:127–36. 25615422 10.1016/j.canlet.2015.01.015

[101] Dong Z, Luo Y, Yuan Z, Tian Y, Jin T, Xu F. Cellular senescence and SASP in tumor progression and therapeutic opportunities. Mol Cancer. 2024;23:181. 39217404 10.1186/s12943-024-02096-7 PMC11365203

[102] Jia L, Li H, Sun Y. Induction of p21-dependent senescence by an NAE inhibitor, MLN4924, as a mechanism of growth suppression. Neoplasia. 2011;13:561–9. 21677879 10.1593/neo.11420 PMC3114249

[103] Lin JJ, Milhollen MA, Smith PG, Narayanan U, Dutta A. NEDD8-targeting drug MLN4924 elicits DNA rereplication by stabilizing Cdt1 in S phase, triggering checkpoint activation, apoptosis, and senescence in cancer cells. Cancer Res. 2010;70:10310–20. 21159650 10.1158/0008-5472.CAN-10-2062 PMC3059213

[104] Wang Y, Luo Z, Pan Y, Wang W, Zhou X, Jeong LS, et al. Targeting protein neddylation with an NEDD8-activating enzyme inhibitor MLN4924 induced apoptosis or senescence in human lymphoma cells. Cancer Biol Ther. 2015;16:420–9. 25782162 10.1080/15384047.2014.1003003 PMC4623239

[105] Zhang S, You X, Xu T, Chen Q, Li H, Dou L, et al. PD-L1 induction via the MEK-JNK-AP1 axis by a neddylation inhibitor promotes cancer-associated immunosuppression. Cell Death Dis. 2022;13:844. 36192389 10.1038/s41419-022-05292-9 PMC9529958

[106] Yoshimura C, Muraoka H, Ochiiwa H, Tsuji S, Hashimoto A, Kazuno H, et al. TAS4464, a highly potent and selective inhibitor of NEDD8-activating enzyme, suppresses neddylation and shows antitumor activity in diverse cancer models. Mol Cancer Ther. 2019;18:1205–16. 31092565 10.1158/1535-7163.MCT-18-0644

[107] Muraoka H, Yoshimura C, Kawabata R, Tsuji S, Hashimoto A, Ochiiwa H, et al. Activity of TAS4464, a novel NEDD8 activating enzyme E1 inhibitor, against multiple myeloma via inactivation of nuclear factor kappaB pathways. Cancer Sci. 2019;110:3802–10. 31583781 10.1111/cas.14209 PMC6890451

[108] Ochiiwa H, Ailiken G, Yokoyama M, Yamagata K, Nagano H, Yoshimura C, et al. TAS4464, a NEDD8-activating enzyme inhibitor, activates both intrinsic and extrinsic apoptotic pathways via c-Myc-mediated regulation in acute myeloid leukemia. Oncogene. 2021;40:1217–30. 33420360 10.1038/s41388-020-01586-4 PMC7892340

[109] Yamamoto N, Shimizu T, Yonemori K, Kitano S, Kondo S, Iwasa S, et al. A first-in-human, phase 1 study of the NEDD8 activating enzyme E1 inhibitor TAS4464 in patients with advanced solid tumors. Invest N. Drugs. 2021;39:1036–46. 10.1007/s10637-020-01055-5 PMC8279981 33560503

[110] Leung CH, Chan DS, Yang H, Abagyan R, Lee SM, Zhu GY, et al. A natural product-like inhibitor of NEDD8-activating enzyme. Chem Commun (Camb). 2011;47:2511–3. 21240405 10.1039/c0cc04927a

[111] Li X, Yokoyama NN, Zhang S, Ding L, Liu HM, Lilly MB, et al. Flavokawain A induces deNEDDylation and Skp2 degradation leading to inhibition of tumorigenesis and cancer progression in the TRAMP transgenic mouse model. Oncotarget. 2015;6:41809–24. 26497688 10.18632/oncotarget.6166 PMC4747190

[112] Li X, Pham V, Tippin M, Fu D, Rendon R, Song L, et al. Flavokawain B targets protein neddylation for enhancing the anti-prostate cancer effect of Bortezomib via Skp2 degradation. Cell Commun Signal. 2019;17:25. 30885218 10.1186/s12964-019-0338-2 PMC6423783

[113] Pham V, Rendon R, Le VX, Tippin M, Fu DJ, Le TH, et al. Gartanin is a novel NEDDylation inhibitor for induction of Skp2 degradation, FBXW2 expression, and autophagy. Mol Carcinog. 2020;59:193–201. 31782573 10.1002/mc.23140 PMC6946862

[114] Zhong HJ, Liu LJ, Chan DS, Wang HM, Chan PW, Ma DL, et al. Structure-based repurposing of FDA-approved drugs as inhibitors of NEDD8-activating enzyme. Biochimie. 2014;102:211–5. 24657219 10.1016/j.biochi.2014.03.005

[115] Zheng YC, Guo YJ, Wang B, Wang C, Mamun MAA, Gao Y, et al. Targeting neddylation E2s: a novel therapeutic strategy in cancer. J Hematol Oncol. 2021;14:57. 33827629 10.1186/s13045-021-01070-w PMC8028724

[116] Lin X, Sun D, Yang S, Cheng K, Wang X, Meng W, et al. UBE2M forms a positive feedback loop with estrogen receptor to drive breast cancer progression and drug resistance. Cell Death Dis. 2024;15:590. 39138151 10.1038/s41419-024-06979-x PMC11322533

[117] Kim JH, Jung JH, Lee HJ, Sim DY, Im E, Park J, et al. UBE2M drives hepatocellular cancer progression as a p53 negative regulator by binding to MDM2 and ribosomal protein L11. Cancers. 2021;13:4901. 34638383 10.3390/cancers13194901 PMC8507934

[118] Heo MJ, Kang SH, Kim YS, Lee JM, Yu J, Kim HR, et al. UBC12-mediated SREBP-1 neddylation worsens metastatic tumor prognosis. Int J Cancer. 2020;147:2550–63. 32449166 10.1002/ijc.33113

[119] Zhao B, Gao C, Shi D, Mao J, Zhao J, Guo L, et al. Knockdown of Nedd8‑conjugating enzyme UBE2M suppresses the proliferation and induces the apoptosis of intrahepatic cholangiocarcinoma cells. Oncol Rep. 2019;42:2670–9. 31545502 10.3892/or.2019.7327

[120] Li L, Kang J, Zhang W, Cai L, Wang S, Liang Y, et al. Validation of NEDD8-conjugating enzyme UBC12 as a new therapeutic target in lung cancer. EBioMedicine. 2019;45:81–91. 31208947 10.1016/j.ebiom.2019.06.005 PMC6642072

[121] Mamun MAA, Liu S, Zhao L, Zhao L, Li ZR, Shen D, et al. Micafungin: a promising inhibitor of UBE2M in cancer cell growth suppression. Eur J Med Chem. 2023;260:115732. 37651876 10.1016/j.ejmech.2023.115732

[122] Chen YF, Liu RZ, Ying WW, Yang YN, Xiang SF, Shao XJ, et al. Arctigenin impairs UBC12 enzyme activity and cullin neddylation to attenuate cancer cells. Acta Pharm Sin. 2023;44:661–9. 10.1038/s41401-022-00992-6 PMC9958092 36138144

[123] Ma T, Song Q, Cheng B, Guo E, Wang X, Li M, et al. Proapoptotic effect of WS-299 induced by NOXA accumulation and NRF2-counterbalanced oxidative stress damage through targeting RBX1-UBE2M interaction in gastric cancers. Bioorg Chem. 2024;144:107142. 38280358 10.1016/j.bioorg.2024.107142

[124] Li A, Ma T, Wang S, Guo Y, Song Q, Liu H, et al. Discovery of WS-384, a first-in-class dual LSD1 and DCN1-UBC12 protein-protein interaction inhibitor for the treatment of non-small cell lung cancer. Biomed Pharmacother. 2024;173:116240. 38401512 10.1016/j.biopha.2024.116240

[125] Song Z, Gao K, Asmamaw MD, Liu YJ, Zheng YC, Shi XJ, et al. Discovery of the antitumor activities of a potent DCN1 inhibitor compound 383 targeting LSD1 in gastric cancer. Eur J Pharm. 2022;916:174725. 10.1016/j.ejphar.2021.174725 34953802

[126] Kurz T, Chou YC, Willems AR, Meyer-Schaller N, Hecht ML, Tyers M, et al. Dcn1 functions as a scaffold-type E3 ligase for cullin neddylation. Mol Cell. 2008;29:23–35. 18206966 10.1016/j.molcel.2007.12.012

[127] Zhou H, Lu J, Liu L, Bernard D, Yang CY, Fernandez-Salas E, et al. A potent small-molecule inhibitor of the DCN1-UBC12 interaction that selectively blocks cullin 3 neddylation. Nat Commun. 2017;8:1150. 29074978 10.1038/s41467-017-01243-7 PMC5658359

[128] Zhou H, Zhou W, Zhou B, Liu L, Chern TR, Chinnaswamy K, et al. High-affinity peptidomimetic inhibitors of the DCN1-UBC12 protein-protein interaction. J Med Chem. 2018;61:1934–50. 29438612 10.1021/acs.jmedchem.7b01455

[129] Zhou H, Lu J, Chinnaswamy K, Stuckey JA, Liu L, McEachern D, et al. Selective inhibition of cullin 3 neddylation through covalent targeting DCN1 protects mice from acetaminophen-induced liver toxicity. Nat Commun. 2021;12:2621. 33976147 10.1038/s41467-021-22924-4 PMC8113459

[130] Scott DC, Hammill JT, Min J, Rhee DY, Connelly M, Sviderskiy VO, et al. Blocking an N-terminal acetylation-dependent protein interaction inhibits an E3 ligase. Nat Chem Biol. 2017;13:850–7. 28581483 10.1038/nchembio.2386 PMC5577376

[131] Hammill JT, Scott DC, Min J, Connelly MC, Holbrook G, Zhu F, et al. Piperidinyl ureas chemically control defective in cullin neddylation 1 (DCN1)-mediated cullin neddylation. J Med Chem. 2018;61:2680–93. 29547696 10.1021/acs.jmedchem.7b01277 PMC5898815

[132] Hammill JT, Bhasin D, Scott DC, Min J, Chen Y, Lu Y, et al. Discovery of an orally bioavailable inhibitor of defective in cullin neddylation 1 (DCN1)-mediated cullin neddylation. J Med Chem. 2018;61:2694–706. 29547693 10.1021/acs.jmedchem.7b01282 PMC5914176

[133] Kim HS, Hammill JT, Scott DC, Chen Y, Min J, Rector J, et al. Discovery of Novel Pyrazolo-pyridone DCN1 inhibitors controlling cullin neddylation. J Med Chem. 2019;62:8429–42. 31465221 10.1021/acs.jmedchem.9b00410 PMC7228038

[134] Kim HS, Hammill JT, Scott DC, Chen Y, Rice AL, Pistel W, et al. Improvement of oral bioavailability of pyrazolo-pyridone inhibitors of the interaction of DCN1/2 and UBE2M. J Med Chem. 2021;64:5850–62. 33945681 10.1021/acs.jmedchem.1c00035 PMC8159160

[135] Wang S, Zhao L, Shi XJ, Ding L, Yang L, Wang ZZ, et al. Development of highly potent, selective, and cellular activetriazolo[1,5- a]pyrimidine-based inhibitors targeting the DCN1-UBC12 protein-protein interaction. J Med Chem. 2019;62:2772–97. 30803229 10.1021/acs.jmedchem.9b00113

[136] Zhou W, Ma L, Ding L, Guo Q, He Z, Yang J, et al. Potent 5-Cyano-6-phenyl-pyrimidin-based derivatives targeting DCN1-UBE2M Interaction. J Med Chem. 2019;62:5382–403. 31157974 10.1021/acs.jmedchem.9b00003

[137] He ZX, An Q, Wei B, Zhou WJ, Wei BF, Gong YP, et al. Discovery of potent and selective 2-(Benzylthio)pyrimidine-based DCN1-UBC12 Inhibitors for anticardiac fibrotic effects. J Med Chem. 2022;65:163–90. 34939411 10.1021/acs.jmedchem.1c01207

[138] Shafique S, Ali W, Kanwal S, Rashid S. Structural basis for cullins and RING component inhibition: targeting E3 ubiquitin pathway conductors for cancer therapeutics. Int J Biol Macromol. 2018;106:532–43. 28802844 10.1016/j.ijbiomac.2017.08.047

[139] Yu Q, Hu Z, Shen Y, Jiang Y, Pan P, Hou T, et al. Gossypol inhibits cullin neddylation by targeting SAG-CUL5 and RBX1-CUL1 complexes. Neoplasia. 2020;22:179–91. 32145688 10.1016/j.neo.2020.02.003 PMC7076571

[140] Sakata E, Yamaguchi Y, Miyauchi Y, Iwai K, Chiba T, Saeki Y, et al. Direct interactions between NEDD8 and ubiquitin E2 conjugating enzymes upregulate cullin-based E3 ligase activity. Nat Struct Mol Biol. 2007;14:167–8. 17206147 10.1038/nsmb1191

[141] Zheng J, Yang X, Harrell JM, Ryzhikov S, Shim EH, Lykke-Andersen K, et al. CAND1 binds to unneddylated CUL1 and regulates the formation of SCF ubiquitin E3 ligase complex. Mol Cell. 2002;10:1519–26. 12504026 10.1016/s1097-2765(02)00784-0

[142] Lobato-Gil S, Heidelberger JB, Maghames C, Bailly A, Brunello L, Rodriguez MS, et al. Proteome-wide identification of NEDD8 modification sites reveals distinct proteomes for canonical and atypical NEDDylation. Cell Rep. 2021;34:108635. 33472076 10.1016/j.celrep.2020.108635

[143] Zuo W, Huang F, Chiang YJ, Li M, Du J, Ding Y, et al. c-Cbl-mediated neddylation antagonizes ubiquitination and degradation of the TGF-beta type II receptor. Mol Cell. 2013;49:499–510. 23290524 10.1016/j.molcel.2012.12.002

[144] Embade N, Fernandez-Ramos D, Varela-Rey M, Beraza N, Sini M, Gutierrez de Juan V, et al. Murine double minute 2 regulates Hu antigen R stability in human liver and colon cancer through NEDDylation. Hepatology. 2012;55:1237–48. 22095636 10.1002/hep.24795 PMC3298572

[145] Kumar D, Das M, Sauceda C, Ellies LG, Kuo K, Parwal P, et al. Degradation of splicing factor SRSF3 contributes to progressive liver disease. J Clin Invest. 2019;129:4477–91. 31393851 10.1172/JCI127374 PMC6763247

[146] Chen J, Shin JH, Zhao R, Phan L, Wang H, Xue Y, et al. CSN6 drives carcinogenesis by positively regulating Myc stability. Nat Commun. 2014;5:5384. 25395170 10.1038/ncomms6384 PMC4234183

[147] Peng Z, Fang W, Wu B, He M, Li S, Wei J, et al. Targeting Smurf1 to block PDK1-Akt signaling in KRAS-mutated colorectal cancer. Nat Chem Biol. 2025;21:59–70. 39039255 10.1038/s41589-024-01683-5

[148] Watson IR, Blanch A, Lin DC, Ohh M, Irwin MS. Mdm2-mediated NEDD8 modification of TAp73 regulates its transactivation function. J Biol Chem. 2006;281:34096–103. 16980297 10.1074/jbc.M603654200

[149] Aoki I, Higuchi M, Gotoh Y. NEDDylation controls the target specificity of E2F1 and apoptosis induction. Oncogene. 2013;32:3954–64. 23001041 10.1038/onc.2012.428

[150] Zhou L, Jiang Y, Liu X, Li L, Yang X, Dong C, et al. Promotion of tumor-associated macrophages infiltration by elevated neddylation pathway via NF-kappaB-CCL2 signaling in lung cancer. Oncogene. 2019;38:5792–804. 31243299 10.1038/s41388-019-0840-4

[151] Zhou L, Lin X, Zhang L, Chen S, Chen J, Zhou Z, et al. Neddylation pathway promotes myeloid-derived suppressor cell infiltration via NF-kappaB-mCXCL5 signaling in lung cancer. Int Immunopharmacol. 2022;113:109329. 36252470 10.1016/j.intimp.2022.109329

[152] Naik SK, Lam EW, Parija M, Prakash S, Jiramongkol Y, Adhya AK, et al. NEDDylation negatively regulates ERRbeta expression to promote breast cancer tumorigenesis and progression. Cell Death Dis. 2020;11:703. 32839427 10.1038/s41419-020-02838-7 PMC7445179

[153] Nikolic I, Cursons J, Shields B, Chappaz S, Sudholz H, Meng X, et al. Enhancing anti-tumor immunity of natural killer cells through targeting IL-15R signaling. Cancer Cell. 2025. 10.1016/j.ccell.2025.05.011 10.1016/j.ccell.2025.05.011 40513576

[154] Watson IR, Li BK, Roche O, Blanch A, Ohh M, Irwin MS. Chemotherapy induces NEDP1-mediated destabilization of MDM2. Oncogene. 2010;29:297–304. 19784069 10.1038/onc.2009.314

[155] Kawamura A, Yoshida S, Aoki K, Shimoyama Y, Yamada K, Yoshida K. DYRK2 maintains genome stability via neddylation of cullins in response to DNA damage. J Cell Sci. 2022;135:jcs259514. 35582972 10.1242/jcs.259514

[156] Tian DW, Wu ZL, Jiang LM, Gao J, Wu CL, Hu HL. Neural precursor cell expressed, developmentally downregulated 8 promotes tumor progression and predicts poor prognosis of patients with bladder cancer. Cancer Sci. 2019;110:458–67. 30407690 10.1111/cas.13865 PMC6317957

[157] Wu K, Chen A, Pan ZQ. Conjugation of Nedd8 to CUL1 enhances the ability of the ROC1-CUL1 complex to promote ubiquitin polymerization. J Biol Chem. 2000;275:32317–24. 10921923 10.1074/jbc.M004847200

[158] Xia X, Hu T, He X, Liu Y, Yu C, Kong W, et al. Neddylation of HER2 inhibits its protein degradation and promotes breast cancer progression. Int J Biol Sci. 2023;19:377–92. 36632463 10.7150/ijbs.75852 PMC9830515

[159] Xie P, Zhang M, He S, Lu K, Chen Y, Xing G, et al. The covalent modifier Nedd8 is critical for the activation of Smurf1 ubiquitin ligase in tumorigenesis. Nat Commun. 2014;5:3733. 24821572 10.1038/ncomms4733

[160] Duda DM, Borg LA, Scott DC, Hunt HW, Hammel M, Schulman BA. Structural insights into NEDD8 activation of cullin-RING ligases: conformational control of conjugation. Cell. 2008;134:995–1006. 18805092 10.1016/j.cell.2008.07.022 PMC2628631

[161] Du MG, Liu F, Chang Y, Tong S, Liu W, Chen YJ, et al. Neddylation modification of the U3 snoRNA-binding protein RRP9 by Smurf1 promotes tumorigenesis. J Biol Chem. 2021;297:101307. 34662580 10.1016/j.jbc.2021.101307 PMC8569593

[162] Xian J, Wang S, Jiang Y, Li L, Cai L, Chen P, et al. Overexpressed NEDD8 as a potential therapeutic target in esophageal squamous cell carcinoma. Cancer Biol Med. 2021;19:504–17. 33733647 10.20892/j.issn.2095-3941.2020.0484 PMC9088192

[163] Chen SM, Lin TK, Tseng YY, Tu CH, Lui TN, Huang SF, et al. Targeting inhibitors of apoptosis proteins suppresses medulloblastoma cell proliferation via G2/M phase arrest and attenuated neddylation of p21. Cancer Med. 2018;7:3988–4003. 29984917 10.1002/cam4.1658 PMC6089189

[164] Liu M, Jiang K, Lin G, Liu P, Yan Y, Ye T, et al. Ajuba inhibits hepatocellular carcinoma cell growth via targeting of beta-catenin and YAP signaling and is regulated by E3 ligase Hakai through neddylation. J Exp Clin Cancer Res. 2018;37:165. 30041665 10.1186/s13046-018-0806-3 PMC6057013

[165] Zhang W, Liang Y, Li L, Wang X, Yan Z, Dong C, et al. The Nedd8-activating enzyme inhibitor MLN4924 (TAK-924/Pevonedistat) induces apoptosis via c-Myc-Noxa axis in head and neck squamous cell carcinoma. Cell Prolif. 2019;52:e12536. 30341788 10.1111/cpr.12536 PMC6496207

[166] Olaizola P, Lee-Law PY, Fernandez-Barrena MG, Alvarez L, Cadamuro M, Azkargorta M, et al. Targeting NAE1-mediated protein hyper-NEDDylation halts cholangiocarcinogenesis and impacts on tumor-stroma crosstalk in experimental models. J Hepatol. 2022;77:177–90. 35217064 10.1016/j.jhep.2022.02.007

[167] Gao Q, Yu GY, Shi JY, Li LH, Zhang WJ, Wang ZC, et al. Neddylation pathway is up-regulated in human intrahepatic cholangiocarcinoma and serves as a potential therapeutic target. Oncotarget. 2014;5:7820–32. 25229838 10.18632/oncotarget.2309 PMC4202163

[168] Shao X, Chen Y, Xu A, Xiang D, Wang W, Du W, et al. Deneddylation of PML/RARalpha reconstructs functional PML nuclear bodies via orchestrating phase separation to eradicate APL. Cell Death Differ. 2022;29:1654–68. 35194189 10.1038/s41418-022-00955-8 PMC9345999

[169] Bravo-Navas S, Yanez L, Romon I, Briz M, Dominguez-Garcia JJ, Pipaon C. Map of ubiquitin-like post-translational modifications in chronic lymphocytic leukemia. Role of p53 lysine 120 NEDDylation. Leukemia. 2021;35:3568–72. 33966047 10.1038/s41375-021-01184-7 PMC8632665

[170] Lee GW, Park JB, Park SY, Seo J, Shin SH, Park JW, et al. The E3 ligase C-CBL inhibits cancer cell migration by neddylating the proto-oncogene c-Src. Oncogene. 2018;37:5552–68. 29899407 10.1038/s41388-018-0354-5

[171] Xiong X, Cui D, Bi Y, Sun Y, Zhao Y. Neddylation modification of ribosomal protein RPS27L or RPS27 by MDM2 or NEDP1 regulates cancer cell survival. FASEB J. 2020;34:13419–29. 32779270 10.1096/fj.202000530RRR

[172] Liu H, Shih YH, Wang WL, Chang WL, Wang YC. UBE1C is upregulated and promotes neddylation of p53 in lung cancer. FASEB J. 2023;37:e23181. 37668436 10.1096/fj.202300629R

[173] Vava A, Paccez JD, Wang Y, Gu X, Bhasin MK, Myers M, et al. DCUN1D1 is an essential regulator of prostate cancer proliferation and tumour growth that acts through neddylation of cullin 1, 3, 4A and 5 and deregulation of Wnt/catenin pathway. Cells. 2023;12:1973. 37566052 10.3390/cells12151973 PMC10417424

[174] Tong S, Si Y, Yu H, Zhang L, Xie P, Jiang W. MLN4924 (Pevonedistat), a protein neddylation inhibitor, suppresses proliferation and migration of human clear cell renal cell carcinoma. Sci Rep. 2017;7:5599. 28717191 10.1038/s41598-017-06098-y PMC5514088

[175] An H, Statsyuk AV. Development of activity-based probes for ubiquitin and ubiquitin-like protein signaling pathways. J Am Chem Soc. 2013;135:16948–62. 24138456 10.1021/ja4099643

[176] An H, Statsyuk AV. An inhibitor of ubiquitin conjugation and aggresome formation. Chem Sci. 2015;6:5235–45. 28717502 10.1039/c5sc01351h PMC5500945

[177] Ma H, Zhuang C, Xu X, Li J, Wang J, Min X, et al. Discovery of benzothiazole derivatives as novel non-sulfamide NEDD8 activating enzyme inhibitors by target-based virtual screening. Eur J Med Chem. 2017;133:174–83. 28388520 10.1016/j.ejmech.2017.03.076

[178] Li Y, Wang C, Xu T, Pan P, Yu Q, Xu L, et al. Discovery of a small molecule inhibitor of cullin neddylation that triggers ER stress to induce autophagy. Acta Pharm Sin B. 2021;11:3567–84. 34900537 10.1016/j.apsb.2021.07.012 PMC8642603

